# Exploring breast tissue microbial composition and the association with breast cancer risk factors

**DOI:** 10.1186/s13058-023-01677-6

**Published:** 2023-07-10

**Authors:** Rana German, Natascia Marino, Chris Hemmerich, Ram Podicheti, Douglas B. Rusch, Leah T. Stiemsma, Hongyu Gao, Xiaoling Xuei, Pam Rockey, Anna Maria Storniolo

**Affiliations:** 1 Susan G. Komen Tissue Bank at the IU Simon Comprehensive Cancer Center, 450 University Blvd, Indianapolis, IN 46202 USA; 2 Hematology/Oncology Division, Department of Medicine, Indiana University School of Medicine, 980 W. Walnut St, R3-C238, Indianapolis, IN 46202 USA; 3 Center for Genomics and Bioinformatics, Indiana University, Bloomington, IN 47405 USA; 4 Natural Science Division, Pepperdine University, Malibu, CA 90263 USA; 5 Department of Medical and Molecular Genetics, Indiana University School of Medicine, Indianapolis, IN 46202 USA

**Keywords:** Normal breast, *Lactobacillus*, *Acetobacter aceti*, *Xanthomonas* sp., *Ralstonia*, Breast cancer risk factors

## Abstract

**Background:**

Microbial dysbiosis has emerged as an important element in the development and progression of various cancers, including breast cancer. However, the microbial composition of the breast from healthy individuals, even relative to risk of developing breast cancer, remains unclear. Here, we performed a comprehensive analysis of the microbiota of the normal breast tissue, which was analyzed in relation to the microbial composition of the tumor and adjacent normal tissue.

**Methods:**

The study cohorts included 403 cancer-free women (who donated normal breast tissue cores) and 76 breast cancer patients (who donated tumor and/or adjacent normal tissue samples). Microbiome profiling was obtained by sequencing the nine hypervariable regions of the 16S rRNA gene (V1V2, V2V3, V3V4, V4V5, V5V7, and V7V9). Transcriptome analysis was also performed on 190 normal breast tissue samples. Breast cancer risk score was assessed using the Tyrer-Cuzick risk model.

**Results:**

The V1V2 amplicon sequencing resulted more suitable for the analysis of the normal breast microbiome and identified *Lactobacillaceae* (*Firmicutes* phylum), *Acetobacterraceae*, and *Xanthomonadaceae* (both *Proteobacteria* phylum) as the most abundant families in the normal breast. However, *Ralstonia* (*Proteobacteria* phylum) was more abundant in both breast tumors and histologically normal tissues adjacent to malignant tumors. We also conducted a correlation analysis between the microbiome and known breast cancer risk factors. Abundances of the bacterial taxa *Acetotobacter aceti*, *Lactobacillus vini, Lactobacillus paracase*i, and *Xanthonomas *sp. were associated with age (*p* < 0.0001), racial background (*p* < 0.0001), and parity (*p* < 0.0001). Finally, transcriptome analysis of normal breast tissues showed an enrichment in metabolism- and immune-related genes in the tissues with abundant *Acetotobacter aceti*, *Lactobacillus vini*, *Lactobacillus paracasei*, and *Xanthonomas *sp.*,* whereas the presence of *Ralstonia* in the normal tissue was linked to dysregulation of genes involved in the carbohydrate metabolic pathway.

**Conclusions:**

This study defines the microbial features of normal breast tissue, thus providing a basis to understand cancer-related dysbiosis. Moreover, the findings reveal that lifestyle factors can significantly affect the normal breast microbial composition.

**Supplementary Information:**

The online version contains supplementary material available at 10.1186/s13058-023-01677-6.

## Background

Risk factors associated with the development of breast cancer (BC) can be classified as either non-modifiable (gender, age, genetic susceptibility) or modifiable (parity, breastfeeding, obesity) [1, 2]. Such factors have been recently associated with variations in gut microbiome. A reduction in *Bacteroides* and increase in *Staphylococcus*, *Enterobacteriaceae* and *Escherichia coli* correlated with high body mass index (BMI) [3]. Furthermore, an independent study reported a strong association between *Streptococcaceae* in the gut and obesity in approximately 600 American adults [4]. Oral and gut microbial differences by racial/ethnic background were also identified, with African Americans showing a higher abundance of Bacteroidetes and a lower abundance of Actinobacteria and Firmicutes as compared with European-Americans [5, 6]. Microorganisms interact with host metabolism and regulate the local microenvironment, thus impacting tissue homeostasis. Several mechanisms linking the microbiome with changes in the tissue microenvironment have been proposed: (1) regulation of innate and adaptive immune responses [7–9]; (2) induction of genomic instability and DNA damage [10]; (3) metabolic activity generating metabolites (estrogens, short chain fatty acids, amino acid, or secondary bile acids) that may promote tumorigenesis or inhibit the growth of pathogenic bacteria [11–13]. Therefore, bacterial communities within a host can be considered an additional environmental factor that may contribute to or be influenced by carcinogenesis.

The breast microbiota, distinct from other body sites, is dominated by the phyla Proteobacteria and Firmicute*s*, likely because of the fatty acid-rich environment in the breast [14]. Recent reports showed differences in the composition and abundance of some specific bacterial taxa between BC patients and healthy individuals experiencing breast augmentation surgeries [10]. Urbaniak et al. reported a higher relative abundance of *Bacillus*, *Enterobacteriaceae* and *Staphylococcus* and a reduction in other bacteria, including *Lactobacillus*, in BC when compared with the histologically normal tissue adjacent to the tumor (NAT) [14, 15]. Another study defined unique microbiota alterations between breast tumor and NAT tissues, with the enrichment of *Methylobacterium radiotolerans* in tumor tissue and *Sphingomonas yanoikuyae* in NAT [16]. Furthermore, genomic sequencing led to the discovery of *Prevotella* in patients with triple-negative BC (TNBC) [17].

Although the breast tumor microbiota has been investigated [8, 10, 15, 18], the bacterial composition of the healthy breast, lacking any disease-associated histological abnormality, remains an underexplored research area. Recently, Hoskinson et al. identified a unique microbial signature associated with BC development (40). This group identified compositional and functional shifts in the microbiome of truly healthy breast tissue relative to normal breast tissue isolated prior to breast cancer diagnosis as well as NAT and tumor tissue (40). Together with transcriptomic and DNA methylation studies [19–22], these reports suggest that a field effect of the tumor on the NAT may occur and the interaction between NAT and tumor may promote the formation of the tumor's microenvironment. Thus, because of the important role of NAT in cancer progression, its use as a reference sample in molecular and microbial studies may limit our ability to identify cancer-related alterations [21–23].

This study aimed to define the microbiota of the disease-free breast tissue and identify the optimal 16S rRNA gene variable region to facilitate this analysis. Special attention was paid to the analysis of the dysbiosis occurring in association with risk factors for BC (i.e., racial background, age, BMI, menopausal status, parity, genetic predisposition) to determine whether these factors may influence breast microbial composition. Furthermore, the relationship between dysbiosis and host transcriptome alterations was investigated to elucidate the potential direct and indirect effects of breast cancer-related microbiota changes.

## Methods

### Study participants and samples

The study cohort consisted of 403 cancer-free women and 76 breast cancer (BC) patients (Table 1, Additional file 1: Table S1 and S2). The cancer-free cohort, or healthy control (HC), comprised healthy women with median age of 50 years, including 180 premenopausal, 195 postmenopausal, and 28 who underwent uterine ablation (Additional file 1: Table S1). The BC cohort included patients who donated either only tumor biopsies (*N* = 11), only normal tissue adjacent to the tumor mass (NAT, *N* = 41), tumor and NAT (*N* = 20), only distant metastases from BC (Met, *N* = 4), Met and Tumor (N = 1), and Met and NAT (= 4) (Additional file 1: Table S2). Fresh frozen normal breast tissue cores were obtained from the Susan G. Komen Tissue Bank (KTB) at IU Simon Comprehensive Cancer Center (IUSCCC, Institutional Review Board (IRB) protocol #1011003097). Fresh frozen tissue cores from BC patients were obtained from the IUSCCC Tissue Procurement and Distribution Core (IRB protocol #11438). Breast specimens were donated voluntarily upon informed consent. Demographics data are reported in Additional file 1: Table S1 and S2. Samples from subjects receiving antibiotic treatment were excluded from the study. Normal breast specimens were collected as detailed in the KTB website (https://komentissuebank.iu.edu/researchers/sop.php). Briefly, the mammary skin was sterilized and numbed with 10 ccs of 1% lidocaine. A nick incision was made with a sterile scalpel and up to six cores were taken from the upper outer quadrant of the breast using the ATEC Breast Biopsy System (Hologic Inc, Bedford, MA). The tissue cores were then transported to the tissue processing room and either frozen in liquid nitrogen within 10 min and subsequently stored at − 195 °C or fixed in formalin or PAXgene. For each sample, an hematoxylin and eosin-stained section from a paraffin-embedded tissue core was analyzed by a pathologist. All the normal specimens used for this analysis lacked clinical and histological breast abnormalities and included a relatively high epithelial content (cellularity > 40%). Lifetime risk of developing BC was estimated by using the Tyrer-Cuzick risk score (IBISv8) as previously described [24, 25]. A threshold of 20% was used to separate high- (> 20%, *N* = 54) from average-risk (≤ 20%, *N* = 349) individuals.

**Table 1:** Demographics of the heathy and breast cancer cohorts

	Healthy	Breast cancer
Total *N*	403	76
Age (median)	50	56
Menopausal status (%)		
Pre	180 (44%)	24 (42%)
Post	195 (49%)	52 (68%)
Uterine ablation	28 (7%)	
Body mass index (%)		
< 30	136 (34%)	11 (15%)
≥ 30	267 (66%)	17 (22%)
N.A		48 (63%)
Racial background (%)		
Caucasian	294 (73%)	59
African American	78 (20%)	13
Asian	22 (5%)	1
Other/N.A	9 (2%)	3
Hispanic (%)	24 (6%)	2
Parity (%)		
Nulliparous	21 (5%)	N.A
Parous	382 (95%)	N.A
Age at menarche (%)		
< 12	84 (20%)	N.A
≥ 12	319 (80%)	N.A
Ever breastfed	358 (89%)	N.A
Ever smoker (%)	88 (22%)	N.A
Tyrer-Cuzick score > 20	54	N.A
Prior treatment (chemo, Surgery)	28 (7%)	35 (56%)
Developed breast cancer later	24 (6%)	N.A
Tumor biomarkers		
ER positive	N.A	47 (62%)
PR positive	N.A	40 (52%)
HER2 positive	N.A	26 (35%)
Tumor grade		
1	N.A	8
2	N.A	25
3	N.A	18
Tumor histology		
IDC	N.A	17
ILC	N.A	2
DCIS	N.A	2
LCIS	N.A	1
AC	N.A	1
C	N.A	2

*ER* estrogen receptor, *PR* progesterone receptor, *HER2* human epidermal growth factor receptor 2, *AC* adenocarcinoma, *C* carcinoma, *DCIS* ductal carcinoma in situ, *LCIS* lobular carcinoma in situ, *ILC* infiltrating lobular carcinoma, *IDC* invasive ductal carcinoma

To control for possible environmental microbial contamination in the breast specimens, a specimen container filled with 5 ml of sterile saline or water was left open in the operating room during breast surgery. Moreover, a second container with sterile water was used to quickly wash gloves and tools (forceps) upon the procedure. Since tissue specimens were taken in different times and locations over the past 12 years by the KTB, it was not possible to get environmental controls retroactively from all locations and, therefore, the environmental controls that were collected on November 2019 at the IUSCCC event were used to represent a typical harvesting and processing room. In addition, negative controls accounting for the kit buffers and columns were included. These environmental controls were stored at − 80 °C and processed in parallel with tissue specimens (Additional file 1: Table S3). Furthermore, QIAseq 16S/ITS Smart Control was used as a positive control for library construction steps.

### Bacterial DNA extraction

Each tissue core (80–150 mg) was divided in two pieces using a sterile scalpel and one portion was processed for bacterial DNA extraction. Tissue processing was performed under sterile conditions and the laminar flow hood (AirScience, PCR-36), the pipettes, and sterile disposable material (filter tips’ boxes, petri dishes and knives) were sprayed with 100% ethanol followed by 30 min UV prior to their use. Semiautomatic purification of the microbial DNA was performed by using the QIAcube Connect robotic workstation (Qiagen, Germantown, MD, cat#9245208) and the QIAamp DNA Microbiome kit (Qiagen, cat# 51704) following a slightly modified version of the manufacturer’s instructions. The protocol included the following steps: Tissue homogenization. The tissue core was added to 2-ml sample tubes RB (Qiagen, cat# 99038) with 500 µl PBS buffer + 500 µl AHL buffer + 35 μl 1 M DTT + 70 µl proteinase K. Tubes were placed in Thermomixer R (Eppendorf, Enfield, CT), and incubated 1 h at 56 °C and 600 rpm speed, and, after a centrifugation step at 10,000×*g* for 10 min, the supernatant was carefully removed. Next automatic DNA extraction was performed on the QIAcube Connect instrument. Host DNA degradation. A total of 190 μl Buffer RDD and 25 μl diluted Benzonase (1:10) were added to the pellet. After mixing, the tubes were incubated at 37 °C for 30 min at 600 rpm. Proteinase K (20 μl) was added and samples were incubated at 56 °C and 600 rpm for 30 min. A total of 200 μl Buffer ATL was added. The mixture was then transferred manually under laminar hood into Pathogen Lysis Tubes. Lysis of bacterial cells. The tubes were placed into BeadBug instrument (Benchmark, cat# D1030) for bead beating. A speed of 4,000 rpm was applied twice for 45 s each time with a 90 s interval between beatings. Tubes were centrifuged at 10,000×*g* for 1 min. Supernatant was transferred manually under laminar hood into 1.5-ml tubes and placed in the QIAcube Connect instrument again for the second part of the protocol. A total of 40 μl Proteinase K was added followed by incubation at 56 °C and 600 rpm for 30 min. Then, 200 μl Buffer APL2 was added followed by an incubation at 70 °C for 10 min. DNA binding. A total of 200 μl 100% ethanol was added to the lysates and the mixture was applied onto QIAamp UCP Mini Columns and centrifuged at 6000×*g* for 1 min. DNA wash and elution. DNA on the columns was washed with 500 μl Buffer AW1 followed by centrifugation and 500 μl Buffer AW2 and centrifugation. DNA was then eluted with 30 µl of AVE Buffer.

### 16S rRNA sequencing

Bacterial DNA samples were submitted to the Center for Medical Genomics at Indiana University where they were quantified using the Qubit dsDNA broad-range assay (Thermo Scientific). Libraries were created using the QIAseq 16S/ITS 96-Screening Panel (Qiagen), which included primers covering all nine hypervariable regions of the 16S rRNA (V1V2, V2V3, V3V4, V4V5, V5V7, and V7V9). The primers are listed in Additional file 1: Table S4. The libraries were generated separately and then quantified on a Bioanalyzer DNA 1000 chip (Agilent Technologies, Santa Clara, CA, USA), and normalized to 2 nM. Once pooled, denatured, and diluted to a final concentration of 10 pM, the libraries were sequenced with an Illumina MiSeq (San Diego, CA, USA). The variable regions targeted by the amplicons ranged in size from 200 to 300 bp.

The pooled samples were demultiplexed using Cutadapt 3.4 [26] from within QIIME2 2021.4 (https://qiime2.org) (28) to separate reads based on the variable region primers. Then, Divisive Amplicon Denoising Algorithm (DADA) 2 [27] was used for quality filtering, sequence denoising, and calling amplicon sequence variants (ASVs). DADA2 was run with the parameters "-p-trunc-len-f 200 -p-trunc-len-r 195" meaning the forward reads was trimmed to 200 bp and the reverse read was trimmed to 195 bp before the forward and reverse reads were merged. Chimeric ASVs were identified and removed using the uchime-denovo command from the QIIME2 VSEARCH [28] plugin. Remaining ASVs were classified using the 138.1 released of the SILVA SSU database [29]. In both the samples and negative controls, 1.6%-7% (based on the variable region) of the ASVs were assigned to the domain Eukaryota (human mitochondria) and were thus excluded in downstream analyses (Additional file 1: Table S5). Moreover, removal of the contaminating taxa based on their higher frequency in low-concentration samples and prevalence in negative control samples was performed using decontam (https://github.com/benjjneb/decontam) as previously described [30]. Violin plots prior to and after this decontamination step were generated using the Seaborn Python package [31] and are shown in Additional file 2: Fig S1. Next, alpha-diversity and beta-diversity were calculated, correlated with sample metadata, and plotted using QIIME2 (https://view.qiime2.org/). Operational Taxonomic Units (OTU) were then generated at 97% sequence similarity using AbundantOTU + 0.95b [32] and used for the graphical representation of the data.

The sequence data were deposited in the National Center for Biotechnology Information (NCBI)-Sequence Read Archive (SRA) under the accession number PRJNA867176.

### RNA extraction and transcriptome analysis

The second piece of the frozen normal breast tissue cores was processed for RNA extraction. First the tissues were homogenized as previously described [24]. Briefly, the tissue was placed into 2-ml prefilled tubes containing 3 mm zirconium beads (Benchmark Scientific, cat.# D1032-30), 350 µl Lysis Buffer and 2-Mercaptoethanol, and were homogenized on BeadBug 6 homogenizer (Benchmark Scientific) in a cold room at the following conditions: 4000 rpm for 45 s was repeated twice with 90 s rest time. Then, the samples were loaded into the QIAcube Connect workstation and total RNA was extracted using the AllPrep DNA/RNA/miRNA kit (Qiagen). The RNA concentration and quality were assessed using Agilent 2100 Bioanalyzer. A RIN (RNA Integrity Number) ≥ six is required to pass the quality control. Of the total RNA samples, 190 samples passed the quality control step and were submitted for sequencing. cDNA library was prepared using the KAPA RNA HyperPrep kit (Roche, Wilmington, MA) on a BiomeK Automated workstation (Beckman coulter, Indianapolis, IN) and sequenced using Illumina NovaSeq 6000. Sequenced reads were adapter trimmed and quality filtered using Trimmomatic ver. 0.38 [33] setting the cutoff threshold for average base quality score at 20 over a window of 3 bases, excluding the reads shorter than 20 bases post-trimming (parameters: LEADING:20 TRAILING:20 SLIDINGWINDOW:3:20 MINLEN:20). Cleaned reads were mapped to Human genome reference sequence GRCh38.p12 with gencode v.28 annotation, using the RNA-seq aligner, STAR version STAR_2.7.3a [34]. Read pairs mapping concordantly and uniquely to the eon regions of the annotated genes were counted using featureCounts tool ver. 2.0.0 [35] of subread package. Read alignments to antisense strand, or to multiple regions on the genome or those overlapping with multiple genes were ignored (parameters: -s 2 -p -B -C). Differential expression analysis was performed using DESeq2 ver. 1.24.0 [36] and the *p*-values were corrected for multiple-testing using the Benjamini–Hochberg method. False discovery rates (FDR) were generated. Transcriptome profiles were divided based on the bacterial abundance (OTU) into high (above the average OTU value) and low (below average OTU value) group. To account for batch effect, two sample batches were analyzed separately, and the resulting data were then merged to generate the final list of differentially expressed genes (DEGs). Pathway analysis was performed by interrogating the Kyoto encyclopedia of genes and genomes (KEGG, https://www.genome.jp/kegg/); because of the absence of DEGs in the *Acetobacter aceti* analysis at FDR < 0.05, a threshold of *p*-value = 0.01 was used to build paths and nodes [37]. Molecular network was created using STRING (https://string-db.org/).

The RNA-seq data were submitted to gene Expression Omnibus (GEO) under accession number GSE205725.

### Statistical analysis

Data are presented as mean ± standard deviation (SD), and statistical significance was analyzed using GraphPad Prism 9 (GraphPad LLC, San Diego, CA, USA). Comparisons of means between two groups or among more than three groups were analyzed using two-tailed Student’s *t*-test and one-way analysis of variance, respectively. Differences were considered statistically significant at *p* < 0.05. Shannon diversity indexes between the various amplicons were compared via pairwise Kruskal–Wallis test. Differentially expressed genes between high- and low-bacterial abundance samples were selected based on the Benjamini–Hochberg adjusted *p*-values. Correlation between bacterial abundance and either BC risk factors or transcriptome profiling was determined via Pearson’s correlation analysis.

## Results

### Study cohort: sample preparation

Fresh frozen breast tissue cores from 403 cancer-free women and 76 BC patients (31 Tumor, 61 NAT, 9 Met) were processed for microbiome analysis. As previously reported, the study of microbiome in low biomass tissue, such as the breast, required both specific technical procedures and bioinformatics analyses to address two types of potential contaminants: environmental contamination from other bacteria and host-derived amplification which may produce higher number of reads than those generated by the limited microbial population [38]. In the processing of the fresh frozen breast tissue cores donated by either healthy or cancer-bearing women, every possible effort was made to preserve sterility, including the use of gloves, face mask, lab coat, laminated biological cabinet, single-use tubes and tips, UV-mediated sterilization of the DNA extraction processor, and alcohol decontamination of any surface [38]. Moreover, to account for any potential environmental contaminants, several negative controls were used including water samples collected in the biopsy operating room, controls for the DNA extraction kit and for the processor (Additional file 1: Table S3). These control samples were included in each extraction group to account for the batch effect. Although sterile procedures were adopted, post-sequencing data analysis revealed the presence of human mitochondrial ASVs in both samples and negative controls, especially under 16S V3V4 region amplification (Additional file 2: Fig S1A, B). This region therefore was removed from further analysis. All amplified regions also contained bacteria-specific reads in the negative control samples. To address these limitations, two phase of data decontamination were performed. First the human ASVs were removed; then, by employing the methods described by Davis et al. [30], most of the environmental contaminants were eliminated (Additional file 2: Fig S1C). Nevertheless, as shown in the heatmap in Additional file 2: Fig S2, bacterial-related reads were still detected in the negative control samples.

### The analysis of low biomass breast tissue generates high background signal

The presence of high levels of bacterial DNA in no-template controls, mostly matching with general contaminants, was also described in previous reports [38]. To assess for potential cross-contamination between samples, OTU detected in the negative controls (Neg CTR) were taxonomically characterized at both family and genus scale. At the family level, the microbial community in the Neg CTR was mostly dominated by Burkholderiaceae (26%), Propionibacteriaceae (4–11%), Comamonadaceae (5–8%), Bacillaceae (5%) and other taxa previously described as general environmental contaminants [37] (Additional file 2: Fig S3). At the genus level, Burkholderia (10.5–32%), Cutibacterium (4.2–11.8%), Streptococcus (2.5–3.5%), and Bacillus (3–3.1%) were found to be abundant in the Neg CTR group (Additional file 2:Fig S4). Moreover, as mentioned in the Methods section, various Neg CTRs were used during each DNA extraction step and included the following conditions: extraction buffer (EB, *N* = 30), extraction room and gloves (ERG, *N* = 4), harvesting room (HR, *N* = 7), harvesting room gloves (HRG, *N* = 10), homogenization buffer (HB, *N* = 26), processing forceps (PF, *N* = 6), processing room (PR, *N* = 6), library internal control or smartcontrol (SC, *N* = 13). We examined the contribution of each Neg CTR type at family and genus level (Additional file 2: Fig S3 and S4), revealing how the microbial contaminant is different in the several Neg CTRs, with the HR and HRG displaying the higher and the SC the lower OTU values, thus suggesting that the inclusion of the appropriate controls is critical for identifying all the environmental contaminants.

### Variant regions in normal and tumor breast tissue

**Fig. 1:**
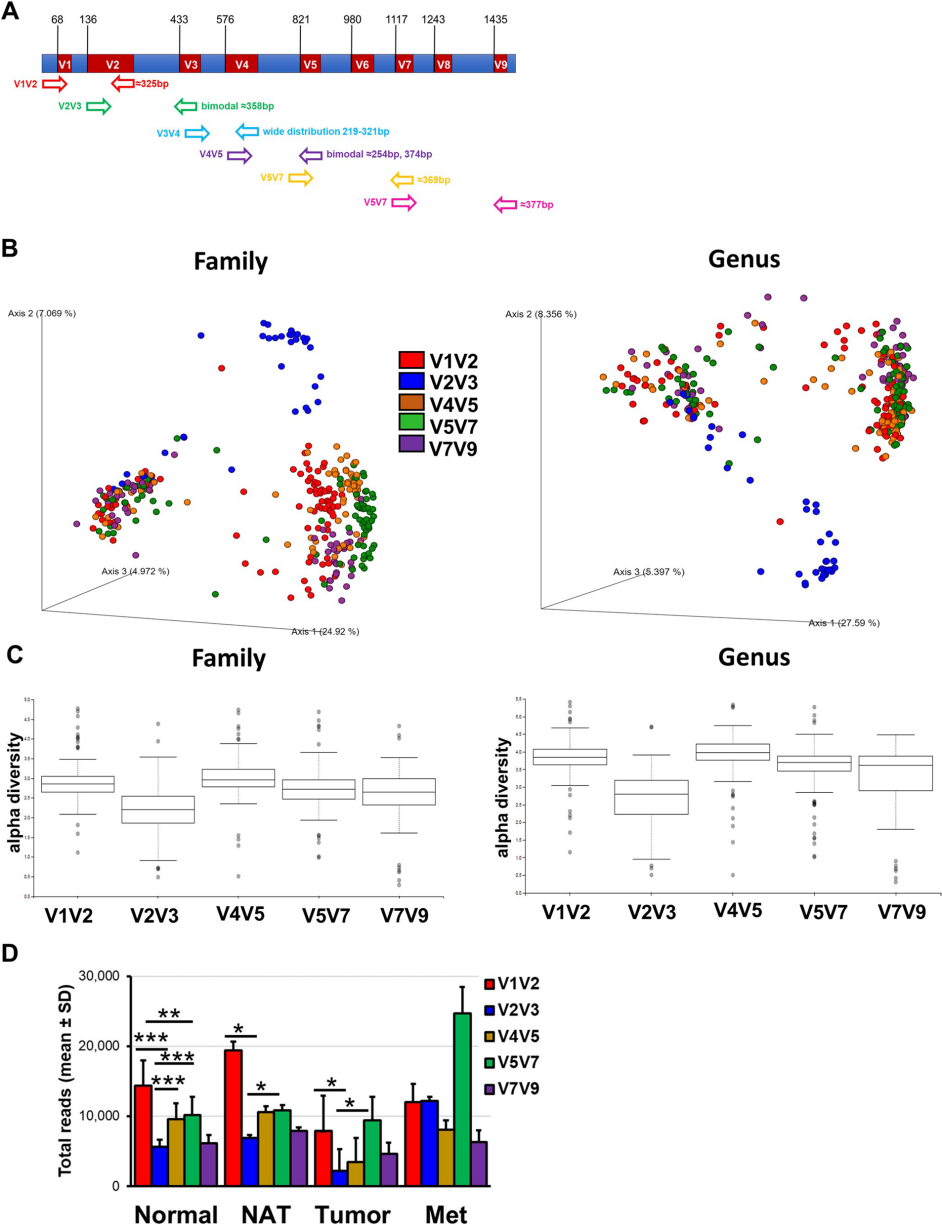
Variable regions analysis of the breast tissue. **A** Microbiome analysis of the breast was performed using primers covering all nine hypervariable regions of the 16S rRNA (V1V2, V2V3, V3V4, V4V5, V5V7, and V7V9). **B** Principal component analysis based on Bray–Curtis distance illustrating the differences between bacterial communities among 40 representative Normal samples via the indicated hypervariable regions at both family and genus level. **C** Shannon diversity index for each of the hypervariable region was determined at both family and genus level. **D** Total read values from the sequencing of each indicated hypervariable region for normal (Normal), normal adjacent to the tumor (NAT), tumor breast tissues (Tumor), and breast cancer metastasis (Met) samples. Nonparametric two-tailed Mann–Whitney U test was used for statistical analysis. *p < 0.05; **p < 0.001; ***p < 0.0001

Sequencing of the 16S rRNA gene is a common approach for the identification, classification, and quantitation of microbial species (Fig. 1A, [39]). Existing studies on breast tissue microbiota have applied different detection methods analyzing different variable regions [10, 40]. We examined amplicons that together covered all nine regions to determine any difference in microbial detection accuracy and efficiency within the normal breast tissues and between normal and tumor tissues (Fig. 1, Additional file 1: Table S3). Thus, the taxonomy of Normal (*N* = 403), NAT (*N* = 61), Tumor (*N* = 31), and Metastatic (Met, *N* = 9) tissues was performed on reads obtained from the sequencing of five amplicons covering all hypervariable regions of the pca.

We performed a principal component analysis (PCA) based on Bray–Curtis distance illustrating the differences between bacterial communities of the normal samples at both family and genus level by each amplified region (Fig. 1B). The PCA revealed three clusters, two of which indicate a major difference in the microbial composition within the Normal group present in all the amplified regions, except for those generated by V2V3, which instead formed a third cluster. The microbial diversity for each of the five amplified regions was calculated at both the family and genus level (Fig. 1C). The Shannon alpha diversity obtained from V2V3 was significantly lower than that of the other four regions (Family: vs V1V2 *p* = 7.2E−08, vs V4V5 *p* = 2.7E−07, vs V5V7 *p* = 6.1E−05, and vs V7V9 *p* = 7.4E−03; Genus: vs V1V2 *p* = 1.2E−10, vs V4V5 *p* = 2.8E−09, vs V5V7 *p* = 5.8E−07, and vs V7V9 *p* = 5.1E−04). After normalization and decontamination, mean read counts obtained from the Normal (median: 9,178 ± 3,524), NAT (median: 11,116 ± 4,925), Tumor (median: 5,496 ± 3,024), and Met (median: 12,629 ± 7,186) samples were not significantly different (two-tailed *t*-test *p* > 0.05). However, when comparing the total read count by amplified region, the V2V3 generated the lowest number of reads in all the experimental groups (Fig. 1D). This could be also a result of sequencing bias, or competition within the Qiagen library prep process. Nevertheless, because of the lack of clustering with the other regions and the lower microbial diversity, the V2V3 dataset was excluded from further analysis.

### Normal breast is enriched in *Lactobacillaceae*, *Acetobacterraceae*, and *Xanthomonadaceae* families

**Fig. 2:**
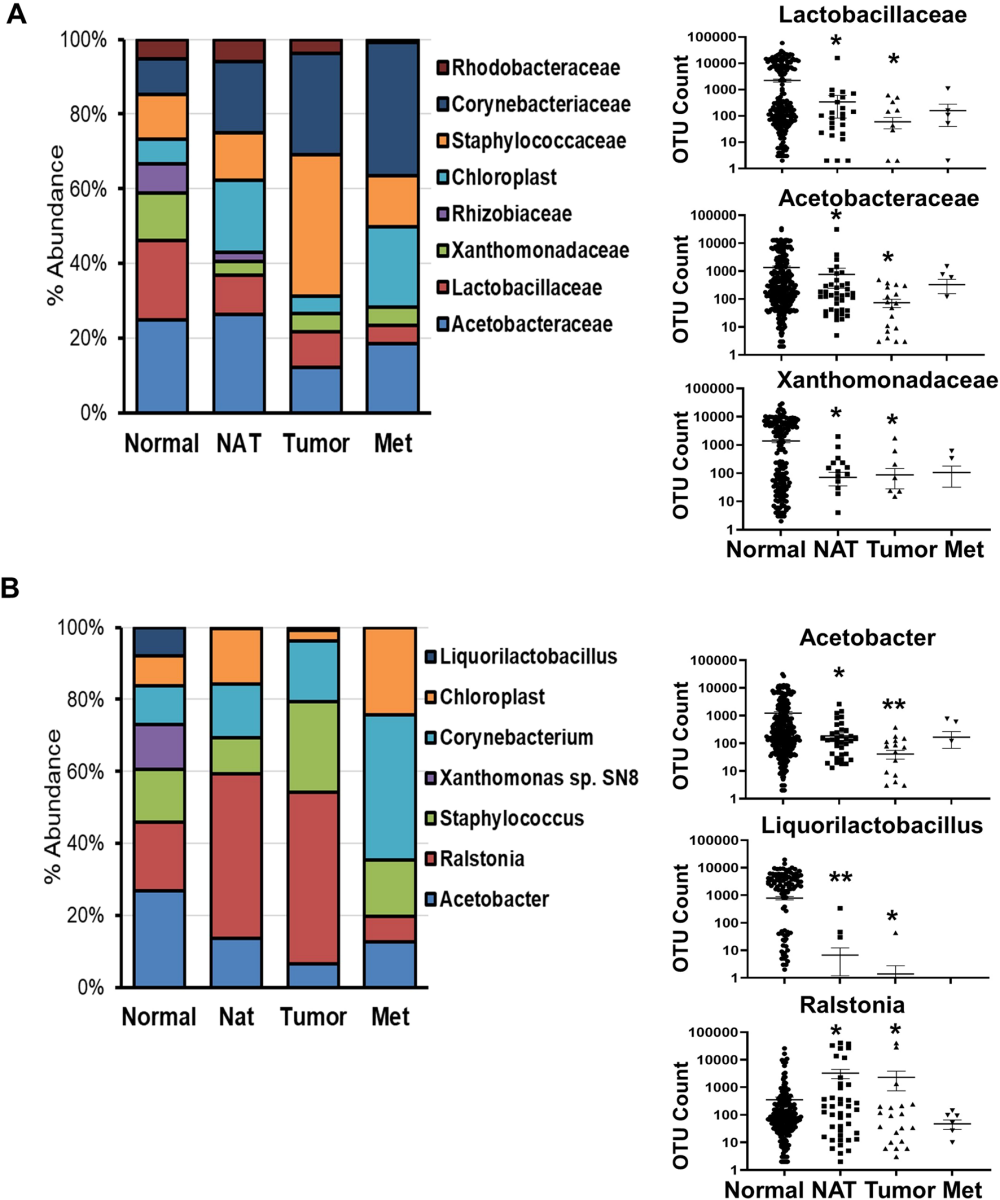
Bacterial abundance in breast tissues. Bacteria taxonomy was examined in the normal (Normal), adjacent normal (NAT), tumor breast tissues (Tumor) and breast cancer metastases (Met) at either family (**A**) and genus (**B**) level. Data from the V1V2 amplified region are shown. Most abundant bacteria (≥ 2% abundance) are displayed in the histobar graph on the left, whereas OTU count of the three most abundant bacteria is shown on the right as the scatter plot, where each dot represents a sample (value of 0 are not included because of the logarithmic scale). Nonparametric two-tailed Mann–Whitney U test was used for statistical analysis. *p < 0.05; **p < 0.001

Microbial abundance at family, genus, and species level was evaluated in Normal, NAT, Tumor, and Met samples examining the data generated by the amplification of V1V2, V4V5, V5V7, and V7V9 regions (Additional file 2:  Fig S2). At the family level, among the most abundant bacteria (> 2%) detected in the normal breast, above the level of the Neg CTRs, *Lactobacillaceae* (6.4%, Firmicutes phylum), *Acetobacterraceae* (7.5%), and *Xanthomonadaceae* (3.8%, both Proteobacteria phylum) were consistently enriched in Normal via all variant region-dependent analyses (Fig. 2A, Additional file 2: Fig S5 and Additional file 1: Table S6). Among the most abundant genera (> 2%), high abundance of *Acetobacter* (Proteobacteria phylum) and *Liquorilactobacillus* (Firmicutes phylum) was detected in Normal via the four variant region-related analyses (Fig. 2B, Additional file 2: Fig S6 and Additional file 1: Table S7). Within the microbial family *Lactobacillaceae*, also *Lacticaseibacillus* and *Lentilactobacillus*, although showing an abundance < 2%, were highly represented in Normal tissue as compared with the other sample types (Additional file 1: Fig S7).

We then examined the microbial abundance in NAT at the family level. *Cyanobacteria* (labeled as *Chloroplasts* in SILVA database) and *Corynebacteriaceae* were detected in NAT and enriched (4.2% and 4.1%, respectively) as compared with Normal (2% and 2.8%, respectively), although the difference lacked statistical significance. Independently of the amplified variant region, *Lactobacillaceae* showed a significant lower abundance in the NAT compared with the Normal (V1V2 *p* = 0.02, Fig. 2A; V4/V5 *p* = 0.03; V5V7 *p* = 0.0012; V7V9 *p* = 0.002, Additional file 2:Fig S6). Similarly, *Acetobacterraceae* resulted less abundant in NAT when compared with Normal (V1V2 *p* = 0.043; V4/V5 *p* = 0.24; V5V7 *p* = 0.6; V7V9 *p* = 0.02). The third most abundant bacterial family in Normal, *Xanthomonadaceae*, also showed a limited presence in NAT (V1V2 *p* = 0.003; V4/V5 *p* = 0.05; V5V7 *p* = 0.0008; V7V9 *p* = 0.02). However, no significant difference was detected between NAT and Tumor groups, indicating that NAT is more similar in this respect to tumor than normal tissue (Fig. 2A, Additional file 2: Fig S5). At the genus level, when compared with the Normal group, NAT showed a lower abundance in both *Acetobacter* (V1V2 *p* = 0.01; V4/V5 *p* = 0.2; V5V7 *p* = 0.3; V7V9 *p* = 0.008) and *Liquorilactobacillus* (V1V2 *p* = 0.0006; V4/V5 *p* = 0.0005; V5V7 *p* = 0.0016; V7V9 *p* = 0.01), whereas no significant difference with the Tumor group was detected (Fig. 2B, Additional file 2: Fig S6). Interestingly, *Ralstonia's* (Proteobacteria phylum) abundance was significantly increased in NAT as compared with the Normal samples in the V1V2 (*p* = 0.018, Fig. 2B), V5V7 (*p* = 0.0086), and V7V9 (*p* = 0.0075) analyses (Additional file 2:Fig S6), whereas no significant difference was detected in the V4V5 analysis (Additional file 2:  Fig S6).

Next, the bacterial abundance in Tumor at the family level was evaluated. Although not highly statistically different (*p* > 0.05) as compared with the Normal because of the limited cohort size and high inter-sample variability, the Tumor samples showed a relative enrichment in *Staphylococcaceae* (5.5% vs 3.6% in Normal) and *Corynebacteriaceae* (3.9% vs 2.8% of the Normal). Moreover, a significant lower abundance in *Lactobacillaceae* (V1V2 *p* = 0.009; V4/V5 *p* = 0.007; V5V7 *p* = 0.007; V7V9 *p* = 0.04), Acetobacterraceae (V1V2 *p* = 0.002; V4/V5 *p* = 0.004; V5V7 *p* = 0.002; V7V9 *p* = 0.005) and *Xanthomonadaceae* (V1V2 *p* = 0.02; V4/V5 *p* = 0.02; V5V7 *p* = 0.0005; V7V9 *p* = 0.01) was detected in the Tumor as compared with the Normal group (Fig. 2A and Additional file 2:  Fig S5). At the genus level, similarly to the NAT group, *Acetobacter* (V1V2 *p* = 0.0003; V4/V5 *p* = 0.006; V5V7 *p* = 0.004; V7V9 *p* = 0.003) and *Liquorilactobacillus* (V1V2 *p* = 0.007; V4/V5 *p* = 0.001; V5V7 *p* = 0.007; V7V9 *p* = 0.03) were absent or of low abundance in most Tumor samples, whereas *Ralstonia* was highly abundant when compared with Normal (V1V2 *p* = 0.04; V4/V5 *p* = 0.04; V5V7 *p* = 0.04; V7V9 *p* = 0.03) (Fig. 2B and Additional file 2: Fig S6). *Staphylococcus* also appeared enriched (5.5% vs 3.5% in Normal), but the difference was not statistically significant. Because of the limited sample size, no statistically significant differences were obtained in any of the comparative analyses with the Met group.

Although the four evaluated regions showed similar results, the OTU count from the V1V2 region was higher than that obtained by amplifying the other regions for *Lactobacillaceae* (*p* = 0.04), *Acetobacterraceae* (*p* < 0.0001), and *Xanthomonadaceae* (*p* = 0.02) (Additional file 2: Fig S8). Hence, the V1V2 region was selected for further analysis.

*Acetobacter aceti*, *Lactobacillus paracasei* and *vini*, and *Xanthomonas *sp. are highly abundant in normal breast tissue.

**Fig. 3:**
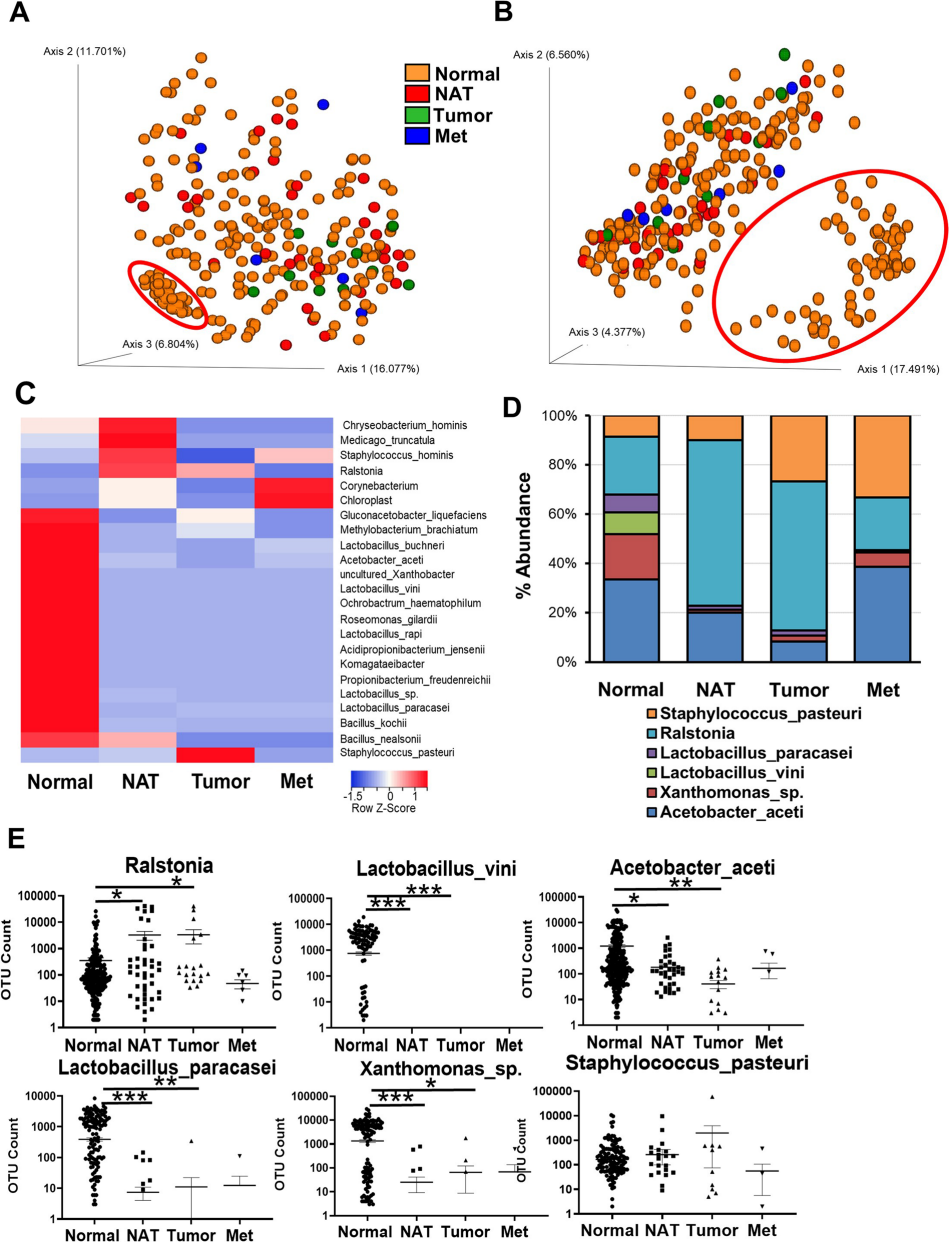
Bacterial abundance in breast tissues at species level. Principal component analysis of Weighted UniFrac (**A**) and Unweighted UniFrac (**B**) plots showing microbiota beta diversity clustering, heat map (**C**) and bar chart (**D**) demonstrating relative abundance of species after 16S rRNA sequencing of healthy (Normal), adjacent normal (NAT), and tumor (Tumor) breast tissues as well as breast cancer metastases (Met). (**E**) Bacterial abundance in Normal, NAT, Tumor and Met samples, each dot represents a sample. Because of the use of the log10 scale values of 0 are not shown. Nonparametric two-tailed Mann–Whitney *U* test was used for statistical analysis. *p < 0.05; **p < 0.001; ***p < 0.0001

A more detailed analysis was performed at species level evaluating the data from the V1V2 region amplification (Additional file 1: Table S8). PCA of Weighted UniFrac and Unweighted UniFrac beta diversity (Fig. 3A, B) revealed overlapping data for the four types of breast tissue, indicating the existence of only small differences between them. However, 55 Normal samples clustered together, separately from the others (circled in red in Fig. 3A, B), due to a high relative abundance of the specific bacterial species described below. The average abundance across Neg CTR was calculated and subtracted from the samples to obtain specific microbial species in the Normal, NAT, Tumor and Met groups (Fig. 3C). The most abundant bacterial species detected in Normal breast tissues was *Acetobacter aceti* at 6.5% abundance, which was reduced to 3.7% in NAT, and to the level of the Neg CTR for the Tumor and Met samples (Fig. 3D). *Xanthomonas *sp., *Lactobacillus vini*, and *Lactobacillus paracasei* resulted highly abundant in the Normal group (3.6%, 1.8%, and 1.4%, respectively), whereas the abundance of these bacteria in the other samples was below 0.6%. *Ralstonia* and *Staphylococcus pasteuri* were, instead, enriched in the Tumor samples (10.4% and 4.6%, respectively) with *Ralstonia* showing a 12.4% abundance also in the NAT samples. Statistical difference in the abundance of these bacterial species was then evaluated in more details.

*Ralstonia* was more abundant in NAT and tumor as compared with Normal (*p* = 0.014, *p* = 0.018, respectively) (Fig. 3E). *Lactobacillus vini* was expressed only in the Normal samples (*p* < 0.001 for both). *Acetobacter aceti* was more abundant in Normal than NAT (*p* = 0.016) and Tumor (*p* = 0.0003). *Lactobacillus paracasei* abundance also was higher in Normal than NAT (*p* = 0.0014) and Tumor (*p* = 0.0007). *Xanthomonas *sp. was more abundant in Normal as compared with NAT (*p* < 0.0001) and Tumor (*p* = 0.01). When examining the OTU values of *Staphilococcus pasteuri*, although the average value in Tumor was greater than that in Normal tissues, no significance difference was detected between the samples.

### Correlation of bacterial abundance with risk factors for BC development

**Fig. 4:**
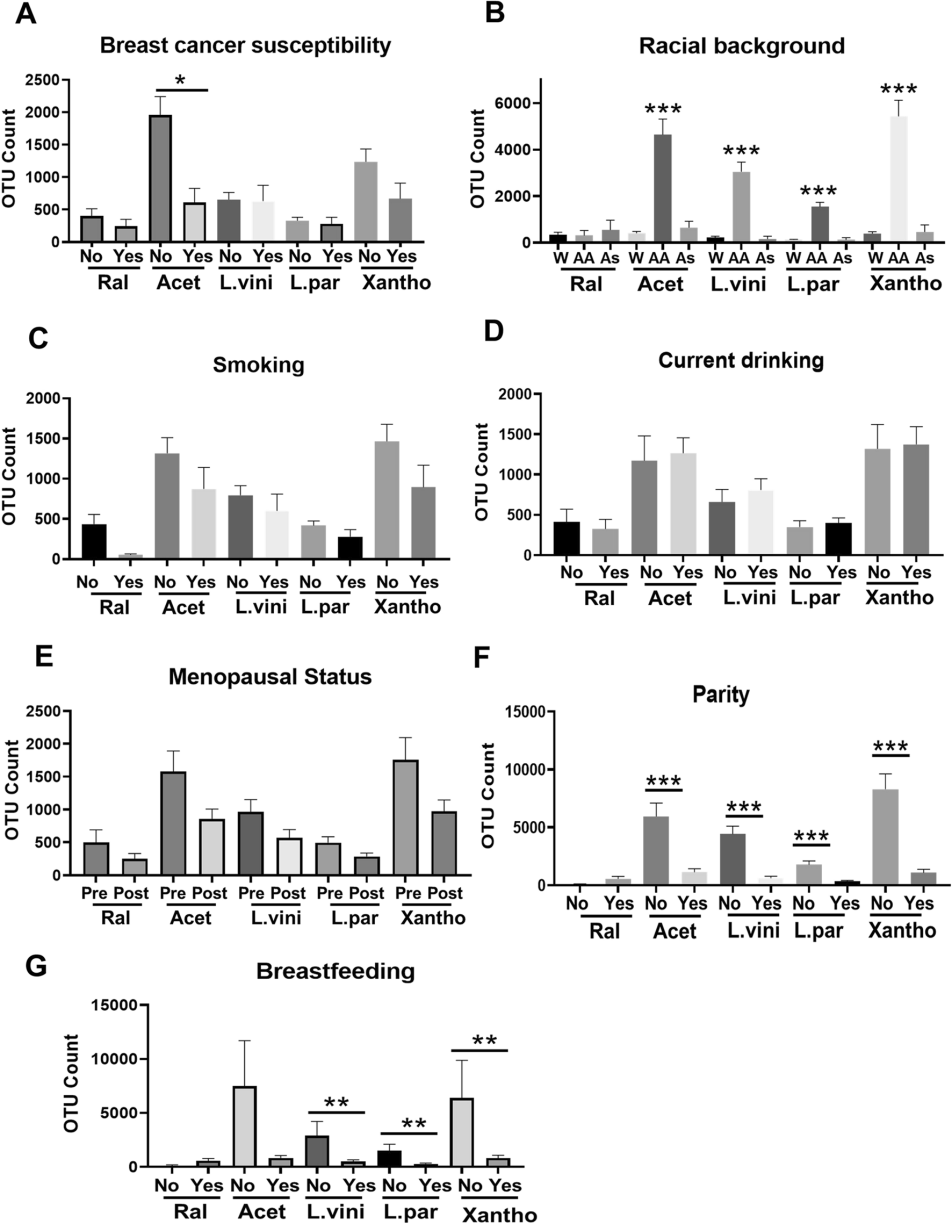
Association of microbial abundance in normal breast tissues with breast cancer risk factors. Pearson’s correlation analysis was performed to examine the link of the abundance of *Ralstonia* (Ral), *Acetobacter aceti* (Acet), *Lactobacillus vini* (L.vini), *Lactobacillus paracasei* (L.par), and *Xanthomonas *sp. (Xantho) with breast cancer susceptibility (**A**), racial background (**B**), smoking (**C**), menopausal status (**D**), parity (**E**) and recent and past breastfeeding (**F**). *p < 0.05; **p < 0.001; ***p < 0.0001

Association of BC risk factors with BC-related dysbiosis has been previously reported [41]. However, the evaluation of such association in cancer-free breast specimens has never been described. Here, we employed Pearson’s correlation analysis to examine the direct or inverse association of risk factors for BC with the abundance, measured as OTU, of *Ralstonia*, *Acetobacter aceti*, *Lactobacillus vini*, *Lactobacillus paracasei*, and *Xanthomonas *sp. in normal breast tissues (Table 2, where bold value indicate *p* < 0.05, and Fig. 4). While none of the detected bacterial species abundance correlates with the Tyrer-Cuzick risk score, the four microbial species abundant in Normal showed an inverse correlation with age (*p* < 0.001), with only *Acetobacter aceti* inversely correlating also with age at first birth (*p* < 0.05; Table 2). No correlation with BMI and age at menarche was observed (Table 2). Interestingly, the same four microbial species identified abundant in Normal highly correlated with one another (*r* = 0.8, *p* < 0.0001, Table 2, complete inter-bacterial correlation analysis in Additional file 1:Table S9) suggesting a potential inter-bacteria interaction [42]. We then examined the abundance of the five bacterial species in relation to predisposition to BC. Normal breast tissues donated by either women with mutation in established BC predisposition genes [*N* = 30, including BRCA1 and BRCA2 mutations (*N* = 15)] or women who donated their biopsy prior to a BC diagnosis (*N* = 24, [20, 43]) were analyzed. Only *Acetobacter aceti* was enriched in the not-predisposed tissue as compared to the breast tissues predisposed to cancer (*p* = 0.04, Fig. 4A). Next, we examined the effect of racial background on the microbial abundance in the normal breast donated by African American (AA, *N* = 78), Asian (*N* = 22), and Caucasian (White, *N* = 294). *Acetobacter aceti*, *Lactobacillus vini*, *Lactobacillus paracasei*, and *Xanthomonas *sp. appeared more abundant in breasts from African American women than those of Caucasian subjects (*p* < 0.0001 for all comparisons, Fig. 4B). We then investigated potential environmental risk factors for BC, such as smoking and drinking. No significant difference in bacterial abundance was observed in relation to either smoking or current alcohol consumption (Fig. 4C, D). Because estrogen levels and breast microenvironment are much different in premenopausal and postmenopausal women, we examined the abundance of these bacteria in relation to menopausal status (Fig. 4E). Although the bacteria appeared slightly more abundant in breasts from premenopausal women, no significant difference was detected between the pre- and postmenopausal groups. Premenopausal women were then evaluated to determine the relation of microbial composition with parity and breastfeeding. *Acetobacter aceti*, *Lactobacillus vini* and *paracasei*, and *Xanthomonas *sp. were abundant in the breasts of nulliparous women as compared to parous subjects (all *p* < 0.0001, Fig. 4F). Furthermore, *Lactobacillus vini* (*p* = 0.003) and *paracasei* (*p* = 0.002), and *Xanthomonas *sp. (*p* = 0.009) were also abundant in the never-breastfed women (*N* = 6) as compared with women who breastfed (*N* = 156) (Fig. 4G).

**Table 2:** Pearson's correlation analysis (*r* coefficient, two-tailed *p*-value)

	TC*	Age	Age at First Birth	Menarche	^#^BMI	*Acetobacter*_*aceti*	*Xanthomonas*_sp.	*Lactobacillus*_*vini*	*Lactobacillus_paracasei*	*Ralstonia*
*Acetobacter_aceti*	− 0.019	− 0.154	− 0.106	− 0.067	− 0.046		0.904	0.824	0.828	− 0.055
	0.706	**0.002**	**0.041**	0.177	0.362		**< 0.0001**	**< 0.0001**	**< 0.0001**	0.265
*Xanthomonas*_sp.	− 0.013	− 0.178	− 0.095	− 0.063	− 0.044	0.904		0.925	0.893	− 0.063
	0.793	**0.0003**	0.065	0.205	0.375	**< 0.0001**		**< 0.0001**	**< 0.0001**	0.207
*Lactobacillus*_*vini*	− 0.022	− 0.178	− 0.084	− 0.057	− 0.050	0.824	0.925		0.938	− 0.061
	0.663	**0.0003**	0.104	0.254	0.313	**< **0.0001	**< 0.0001**		**< 0.0001**	0.220
*Lactobacillus*_*paracasei*	− 0.025	− 0.176	− 0.095	− 0.051	− 0.037	− 0.065	0.893	0.938		− 0.065
	0.624	**0.0004**	0.067	0.312	0.454	0.189	**< 0.0001**	**< 0.0001**		0.189
*Ralstonia*	0.050	− 0.036	0.056	− 0.031	− 0.045	− 0.055	− 0.062	− 0.061	− 0.065	
	0.313	0.474	0.280	0.535	0.367	0.265	0.207	0.220	0.189	

*TC: Tyrer-Cuzick lifetime risk score

^#^BMI: body mass index

### Bacterial abundance in the normal breast correlates with metabolism- and immune-related gene dysregulation in the host tissue

**Fig. 5:**
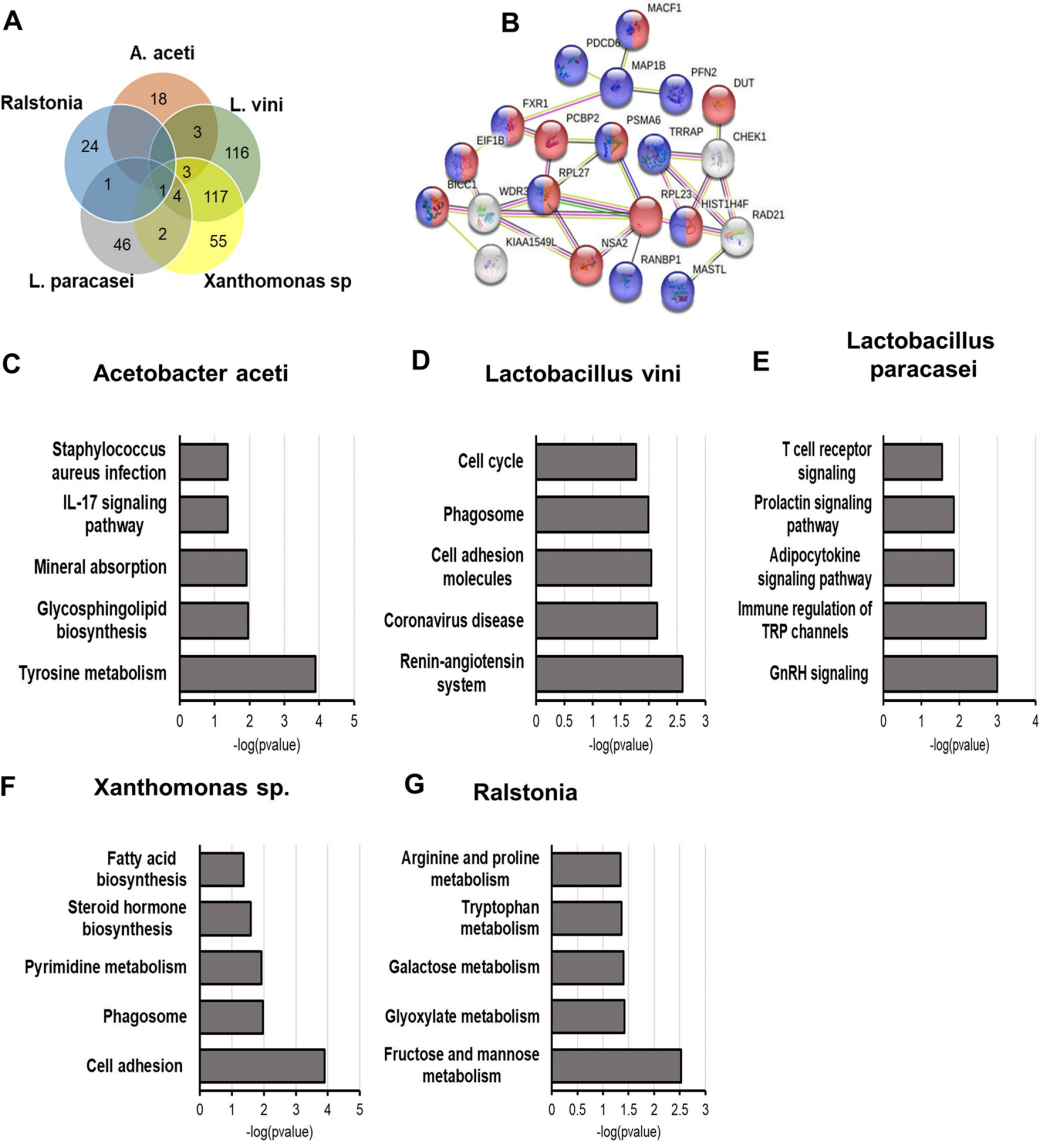
Link between microbial composition and gene enrichment in the breast. Transcriptomic analysis of the normal breast tissues (*N* = 190) was performed. (**A**) Venn diagram shows the common differentially expressed genes linked to the microbial abundance of *Acetobacter aceti* (*A. aceti*), *Lactobacillus vini* (*L. vini*), *Lactobacillus paracasei* (*L. paracasei*), *Xanthomonas* sp, and *Ralstonia.* B) STRING-generated molecular pathway of the genes enriched in both *Lactobacillus vini* and *Xanthomonas* sp. analyses. KEGG pathway analysis was performed for genes enriched in tissues abundant with *Acetobacter aceti* (**C**), *Lactobacillus vini* (**D**), *Lactobacillus paracasei* **(E**), *Xanthomonas *sp. (**F**) and *Ralstonia* (**G**)

The interaction between the microbiome and the host biological processes has been widely reported [7–9, 12–14]. The presence and effects of bacterial communities within a host could be one additional environmental factor with a potential role in the pathophysiological process of breast carcinogenesis. Transcriptomic analysis was performed on the second half of the breast tissue core from 190 Normal samples selected for their best RNA quality and yield (Additional file 1: Table S1). Taking into account any batch effect, differential expression analysis was performed based on the microbial abundance OTU values for *Ralstonia*, *Acetobacter aceti*, *Lactobacillus vini*, *Lactobacillus paracasei*, and *Xanthomonas* sp., and differentially expressed genes (DEGs) between high (OTU values ≥ average) and low (OTU values < average) group were identified (Additional file 1: Table S10). A threshold of FDR < 0.02 was applied for the DEG analysis except for the analysis of gene expression alterations associated with *Acetobacter aceti,* which generated only 22 DEGs at *p* < 0.01 (FDR > 0.2), thus suggesting a limited influence of A*cetobacter aceti* abundance on the tissue homeostasis (Additional file 1: Table S10). Interestingly, the genes disregulated in the *Lactobacillus vini* and *Xanthomonas *sp. groups showed a 51.2%-69.4% overlap, with common genes being mostly involved in acetylation (*p* = 0.003) and RNA binding (*p* = 0.0008) (Fig. 5A, B).

Pathway analysis was then performed using Kyoto Encyclopedia of Genes and Genomes (KEGG) database [37]. Normal tissues abundant in *Acetobacter aceti* were enriched for genes involved in tyrosine metabolism (*p* = 0.03) and IL-17 signaling (*p* = 0.04) (Fig. 5C). Among the DEGs, the highest upregulated gene encoded for the endoplasmic reticulum chaperone protein, CLGN (logFC: 1.29, *p* = 0.0001), whereas the most downregulated gene was KTR16 (logFC:-1.49, *p* = 0.0001), which also inversely correlated with the microbial level in the breast (*r* = − 0.15, *p* = 0.03). Breast tissue abundant in *Lactobacillus vini* displayed an enrichment in genes involved in immune response (KEGG labeled coronavirus disease, *p* = 0.002), cell adhesion (*p* = 0.009), and cell cycle (*p* = 0.01) (Fig. 5D). MTND1P23, HMGB1P6, and CHRM3 resulted highly upregulated (logFC:7.44, *FDR* < 0.0001; logFC:1.54, FDR = 0.0007; logFC:1.44, FDR = 0.0009, respectively) and their expression directly correlated with *Lactobacillus vini* level (*r* = 0.23, *p* = 0.002; *r* = 0.26, *p* = 0.0003; and *r* = 0.27, *p* = 0.0001, respectively). Moreover, an inverse correlation with the expression of NDNF (*r* = − 029, *p* < 0.0001), SCD (*r* = − 0.25, *p* = 0.0006), and MT-TS1 (*r* = − 0.29, *p* < 0.0001) was detected. Gene set enrichment analysis of the DEGs linked to *Lactobacillus paracasei* revealed the alterations of genes related to T cell receptor signaling (*p* = 0.02) and immune regulation of TRP channel (*p* = 0.002) (Fig. 5E). The abundance of *Lactobacillus paracasei* displayed, among other DEGs, the upregulation (logFC: 2.08, FDR = 0.001) as well as direct correlation (*r* = 0.17, *p* = 0.02) with the transmembrane serine protease encoding gene CORIN. *Xanthomonas *sp. abundant tissues showed an enrichment in genes linked with pyrimidine metabolism (*p* = 0.01), fatty acid biosynthesis (*p* = 0.04), and cell adhesion (*p* = 0.0001) (Fig. 5F). *Xanthomonas *sp.-linked DEGs analysis showed the upregulation of MTND1P23 (logFC: 7.5, FDR < 0.0001) and downregulation of NDNF (logFC: − 5.06, *FDR* < 0.0001) and the stearoyl-CoA desaturase encoding gene SCD (logFC: − 6.28, FDR = 0.005). Also, similarly to *Lactobacillus vini*, microbial level correlated directly with HMGB1P6 (*r* = 0.36, *p* < 0.0001) and CHRM3 (*r* = 0.28, *p* = 0.0001) and inversely with MT-TS1 (*r* = 0.28, *p* = 0.0001), NDNF (*r* = − 0.28, *p* < 0.0001), and SCD (*r* = − 0.25, *p* = 0.0005). Metabolic pathways appeared to be linked to the abundance of *Ralstonia* in normal breast and included fructose (*p* = 0.003) and galactose (*p* = 0.03) metabolism (Fig. 5G). In terms of genes, LALBA (logFC: 6.07, *p* < 0.0001), involved in lactose synthesis, was detected as the most upregulated, whereas PRODH (log: − 4.67, *p* = 0.0001) and DOK7 (logFC: − 2.46, *p* = 0.0005) were highly downregulated in *Ralstonia*-abundant normal breast tissues. However, no significant correlation between gene expression and microbial abundance was detected via Pearson analysis.

## Discussion

Although distinct dysbiotic bacterial signatures related to disease states are being increasingly recognized using high throughput sequencing techniques, the microbiome of healthy breast tissue remains underexplored. Here, we characterized the microbiome of the normal breast by examining a large cohort (*N* = 403) of breast tissue biopsies donated by healthy women. We identified bacteria uniquely abundant in the normal tissue as compared with the uninvolved tissue adjacent to tumor (NAT) and tumor and, for the first time, correlated their level with BC risk factors and host transcriptomic changes. Our data also confirmed that, as previously reported [43], NAT, often used as a surrogate for healthy controls, displays bacterial dysbiosis as compared with the normal breast tissue from healthy donors, similarly to what observed in tumor samples.

Recent findings demonstrated the existence of microbiota in internal organs once believed sterile, including the lung, pancreas, and breast [14]. Regarding the source of the microbiota in the breast, several hypotheses have been proposed and investigated including the skin via the nipple-areolar orifices, nipple-oral contact via lactation and/or sexual contact, and, more recently, translocation from the gastrointestinal tract [15, 44]. It is suggested that organ-specific microbiota plays a role in tissue homeostasis, tumor development, and therapeutic resistance [16]. New discoveries in the cancer-related microbiome have been made possible with the use of next-generation sequencing technologies. However, considerable differences in the employed methodologies, with respect to specimen treatment after collection, DNA isolation, target hypervariable region selection for sequencing, and sequence analysis workflows, have resulted in considerable heterogeneity in results, delaying the assessment of the existence of a link between dysbiosis and BC. Inconsistency in the choice of hypervariable region amplified to define the breast tissue microbiota remains a major concern, especially since specific hypervariable regions are more likely to identify certain taxa [45]. In our study of the breast tissue, six amplicons covering the nine 16S rRNA hypervariable regions were examined (V1V2, V2V3, V3V4, V4V5, V5V7, and V7V9). Our findings determined the low specificity of V3V4 and confirmed the data from He et al., showing a difference between the amplified regions in both alpha diversity, with the V2V3 displaying the lowest diversity, and number of reads, with V1V2 generating overall a higher ASV count.

Investigation of low biomass specimens such as the breast tissue, where the microbiota abundance is relatively limited as compared to microbe-rich organs as the guts, is challenging. The analysis of such low biomass tissue needs to consider the impact of external contaminants and experimental artifacts [38]. We used multiple “no template controls” such as storage buffers, elution buffers, or water in our study as good approximates for contaminants introduced during sample collection and storage, extraction and library preparation steps. Hence, our analysis revealed not only the main contaminants (i.e., Burkholderiaceae and Propionibacteriaceae) often reported abundant in BC [46–48], but also that contaminants composition varied in relation to both negative control type and amplicon primers used for the 16S rRNA sequencing. This clearly indicates that appropriate negative control needs to be included in the workflow for microbiome analysis of the breast.

In breast tissue, Proteobacteria and Firmicutes have been reported to be the most abundant phyla, which is distinct from other tissues where these phyla, especially Proteobacteria*,* represent a small portion of the total bacterial load [10, 14, 16, 43]. Analysis of breast tumors and NAT from the same patient showed unique microbial communities associated with tumors, with the high abundance of *Sphingomonas yanoikuyae* in normal tissue and *Methylobacterium radiotolerans* in tumor tissue [16]. Moreover, Banerjee et al. detected a distinct microbial signature associated with TNBC [17, 49]. Urbaniak et al. reported that NAT from women with BC compared to tissue from healthy controls had higher relative abundance of *Bacillus*, *Enterobacteriaceae*, and *Staphylococcus* [10]. However, a study with a Mediterranean population found more similarities than differences between NAT and tumors [50]. A recent publication from Tzeng et al. revealed that tumor tissues contained a much higher percentage of the families *Pseudomonadaceae* and *Enterobacteriaceae* and the genera *Pseudomonas* and *Proteus* [8]. Recently, Hoskinson et al. reported the compositional shifts in bacterial abundance in NAT and tumor tissues as well as breast tissues prior to a clinical manifestation of cancer as compared with healthy breasts [20, 43]. Independent from the findings’ variability, these reports suggest a link between microbial dysbiosis and BC. The variability in the specific bacteria identified can be largely explained by differences in methodology, not only in the care to avoid contaminants, but importantly, in the choice of “normal” controls. In early microbiome investigations, breast tissue obtained from women undergoing reduction mammoplasty were used as poor substitutes for healthy controls [10, 14]. These tissues present an hyperproliferative phenotype [51]. More recently, the histologically normal tissue surrounding the tumor lesions has been used to as the "healthy" control in these experiments [8, 48]. However, multiple recent publications have documented a “field effect” of BC, with histologically normal tissue displaying both genetic and epigenetic aberrations [20–23, 52].

With the exception of the investigation by Hoskinson et al where 49 breast tissue cores from healthy women were examined, our study represents the first large-scale analysis of the microbiota of the normal breast. We detected *Lactobacillaceae* (Firmicutes phylum), *Acetobacterraceae*, and *Xanthomonadaceae* (both Proteobacteria phylum) as more abundant families (> 2%) and *Acetobacter* and *Liquorilactobacillus* as the more abundant genera in the normal breast as compared to both the NAT and tumor samples. These findings further confirmed the biological difference between normal and NAT tissues and, as also reported by Hoskinson et al. [43], the similarity in microbial composition between NAT and tumor tissue.

Four main bacterial species were identified as predominant in the normal breast as compared with the other tissue here analyzed: *Lactobacillus paracasei*, *Lactobacillus vini*, *Acetobacter aceti*, and *Xanthomonas *sp. In exploring the roles of these bacteria in the breast, the literature pointed to possible involvement in metabolic pathways. The genus *Lactobacillus*, including *Lactobacillus paracasei*, was previously reported to be more common in healthy breast tissues than in cancerous tissues and, because of its immunomodulatory effect, may have a role in BC prevention [18, 53]. *Lactobacillus vini*, generally isolated on organic matrices, was detected as member of human fecal microbiota for the first time by Rossi et al. [54]. *Acetobacter aceti* is an aerobic bacterium widespread in sugary, acidic and alcoholic niches. The investigation of this bacterium in human tissues is limited. Aghazadeh et al. showed that a strain of the same genus, *Acetobacter syzygii*, exhibited significant cytotoxicity toward a squamous cell carcinoma cell lines [55], suggesting that, similarly to *Lactobacillus*, this bacterium may also have beneficial properties in the breast. Interestingly, synergistic interaction between *Acetobacter* and *Lactobacillus* was reported in *Drosophila melanogaster* gut and led to nutriments availability modulation with reduction in hist triglyceride [42]. In the Aghazadeh study, a direct correlation between the levels of these bacteria in the normal breast was also observed. The family *Xanthobacteraceae* was reported decreased in abundance in NAT and tumor tissues compared with normal breast from healthy women, thus confirming our data [43]. Also, *Xanthomonas *sp. was recently found abundant (6%) in normal breast of Chinese women [46], but the literature on the role of this microbial species in human is very limited. Although, because of the decontamination approach here employed, the OTU levels were lower than those previously described, consistently with previous reports [18], tumor samples displayed an abundance of *Enterobacteriaceae* (1.2%), *Staphylococcus* (2%), *Corynebacteriaceae* (1.7%), *Corynebacterium* (0.98%), *Anoxybacillus* (3%), *Prevotella* (0.98%), and *Rothia* (0.5%) as compared to the Normal group. Nevertheless, *Ralstonia* (Proteobacteria phylum), almost absent from the normal tissues, showed higher abundance in both NAT and Tumor as compared with the Normal (*p* = 0.014, *p* = 0.018, respectively). This bacterium was previously detected in human milk [56] and silicone breast implant biofilms [57]. Our data confirm previous findings indicating *Ralstonia* as the most dominant bacterial genus in the breast tumor tissues [58–60]; however, we also showed *Ralstonia* presence in the NAT. Interestingly, the fact that other studies report the abundance of *Ralstonia* in the normal breast is explained by their use of NAT as source of normal tissue.

Exogenous and endogenous factors can promote fluctuations in microbial abundance and functions. Many of the important life-style risk factors for cancer like obesity, smoking, diet, and alcohol can also cause perturbations in the microbial composition [41]. Here, we examined the correlation between the abundance of *Ralstonia*, *Acetobacter aceti*, *Lactobacillus vini*, *Lactobacillus paracasei*, and *Xanthomonas *sp. in the normal breast tissues and BC risk factors, such as age, parity, breastfeeding, smoking, alcohol consumption, age at menarche, and body mass index. Moreover, a cohort of 58 breast tissues from women either at genetic risk for BC or who developed BC post-tissue donation [20, 43] was examined with respect to microbial enrichment. As opposed to the work by Tzeng et al., where subtle differences in microbial profiles between healthy control and high-risk tissues were detected [8], no significant difference was observed in our investigation except for *Acetobacter aceti* being less abundant in genetically predisposed breast tissue. *Ralstonia* failed to show correlation with any of the examined risk factors, probably due to its low level in the normal tissue. The Normal-specific bacteria inversely correlated with age, the strongest risk factor for BC, and were enriched in nulliparous women as well as in women who did not breastfeed as compared with those who breastfed. Whether these bacteria, abundant in the normal breast and, except for *Acetobacter aceti*, previously detected in breast milk [61], are lost via breastfeeding requires further investigation. Racial background is another key determinant of BC. While BC incidence overall is higher in Caucasian women, African American women are at a higher risk of developing the more aggressive TNBC. Unexpectedly, *Acetobacter aceti*, *Lactobacillus vini* and *paracasei*, and *Xanthomonas *sp. were enriched in normal breasts from African American women as compared with the Caucasian and Asian cohorts. Interestingly, *Lactobacillus* was previously reported highly abundant in TNBC from White non-Hispanic patients as compared with Black non-Hispanic tumor tissues [58], whereas the family Xanthomonadaceae was abundant in White non-Hispanic tumors [59]. Although previous reports evaluated *Ralstonia* abundance in breasts from Black non-Hispanic and White non-Hispanic women, their results were discordant with our current study supposedly because of their use of NAT as control tissue [58, 59].

Next, to test the hypothesis of a key microbial–host crosstalk that may influence the tumor microenvironment, we examined the transcriptome changes in 190 normal breast tissues and their association with the abundance of *Ralstonia*, *Acetobacter aceti*, *Lactobacillus vini*, *Lactobacillus paracasei*, and *Xanthomonas *sp. Overall, our findings confirm the data from Tzeng et al., where the breast bacteria exhibit significant associations with immunomodulatory genes [8]. The gene set enrichment analysis of the DEGs linked with microbial level in normal breast revealed the involvement of immune pathways including the IL17 signaling (*Acetobacter aceti*), T cell receptor signaling (*Lactobacillus paracasei*), inflammatory response (*Lactobacillus vini*), and phagocytotic process [62] (*Lactobacillus vini* and *Xanthomoas* sp.). Moreover, although the influence of *Acetobacter aceti* on the tissue homeostasis appeared limited, its abundance inversely correlated with keratin 16 (KRT16), a structural protein recently shown to regulate innate immunity in response to epidermal barrier stress [63]. The study by Hoskinson et al., where Spearman’s rank correlation analysis between the host transcriptome and microbial taxa and genes in healthy breast tissues was performed, identified the gene CYP24A1, encoding for 24-hydroxylase, inversely associated with a number of bacterial nutrient transport and metabolic pathways [43]. Similarly, we found the relative abundance of certain microbes in the normal breast from healthy women to be linked with metabolic pathways such as lactose and galactose metabolism as well as fatty acid and cortisol synthesis. Specifically, in our study the abundance of both *Lactobacillus vini* and *Xanthomonas* sp. resulted inversely correlated with SCD expression, which encodes a stearoyl-CoA desaturase involved in fatty acid biosynthesis and whose elevated expression in human BCs predicts poor survival [64]. Interestingly, tissues abundant in *Ralsonia*, in addition to upregulation of carbohydrate metabolism-related genes, presented a significant downregulation in DOK7, which was recently reported to inhibit proliferation, migration, and invasion of BC cells through the PI3K/PTEN/AKT pathway [65]. Overall, our data showed that changes in the expression of metabolism-related genes in the breast, previously linked with breast cancer susceptibility [20, 43, 66], are associated with a switch in microbial composition of the disease-free breast. Thus, our findings suggest that changes in microbial composition, and subsequently in the metabolic milieu of the breast, may occur prior to cancer initiation as a consequence of the exposure to a specific risk factor (i.e., age, and as recently reported [67], racial background). Further mechanistic investigation is required to link these bacterial species to any functional role of this microbial population in heathy breasts.

Overall, our data show a distinctive microbiota for the normal breast tissue, which seem to reduce and even disappear in NAT and, in a greater degree, in tumor. However, no correlation with Tyrer-Cuzick risk score was identified, thus suggesting that dysbiosis in the breast may occur late during the carcinogenesis process and might be a consequence rather than the cause of the changes in the microenvironmental milieu dictated by the already established tumor cells. An exception is given by the recently reported relationship between breast and gut microbiome. The breast microbiota composition can be directly [44] and indirectly [41, 68–70] influenced by alterations of the gut microbiota. Moreover, recently, Parida et al. identified a gastrointestinal pro-oncogenic bacterium, *Bacteroides fragilis*, in breast tumor samples and suggested a potential role in BC initiation [44]*.*

This study bears several limitations, including the lack of microscopic evaluation of breast tissues that would visually establish the presence of bacteria and their abundance in the processed tissue samples as well as the absence of mechanistic insights into the role of the identified microbial species in BC development. In vitro investigation shall follow to address these points. Although our attempts in detecting microbial population in a low biomass tissue such as the normal breast via quantitative PCR and in situ hybridization failed, our findings rely on 16S rRNA analysis, which is a widely accepted approach in the study of the breast microbiome [8, 43, 67]. Moreover, we detected a lack of correlation between the bacteria and BMI, which was unexpected since bacteria can adapt to the fatty acid environment in the breast tissue. Hematoxylin eosin analysis may be a more appropriate approach to elucidating the association of the adipose tissue area in the analyzed biopsy with the microbial level. Finally, microbiota-derived metabolites are crucial mediators of host-microbial interactions [71], therefore metabolomic analysis would be critical for defining the consequences of the dysbiosis.

## Conclusions

**Fig. 6:**
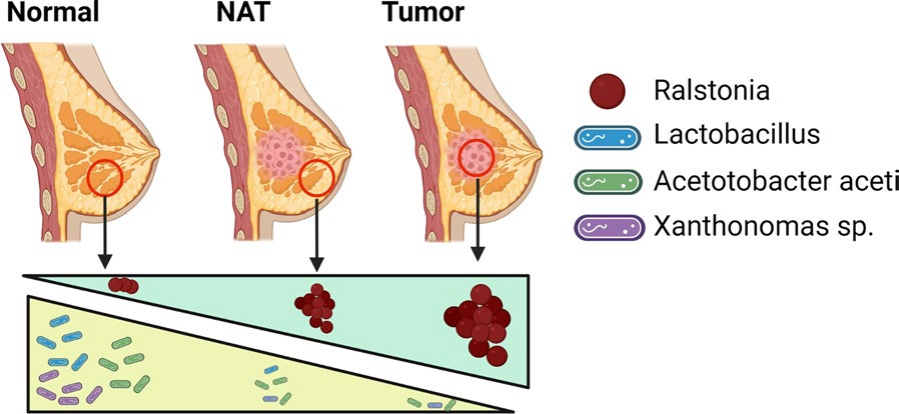
Major microbiome changes in the breast: from healthy to tumor. The normal breast tissue (Normal) shows low to undetectable levels of *Ralstonia*, whereas the tissue adjacent to tumor (NAT), which may represent an intermediate stage between normal and cancer status, and the tumor contain increasing level of this bacterial species. The opposite trends is observed for *Lactobacillus*, *Acetobacter aceti*, and *Xanthomonas *sp., which appear highly abundant in normal breast tissue and scarce in NAT and tumor

In summary, this investigation revealed for the first time the presence of *Acetobacter aceti*, *Lactobacillus vini* and *paracasei*, and *Xanthomonas *sp. in the normal breast, whereas *Ralstonia* was found abundant in NAT and tumor (Fig. 6). Our findings clearly have shown differences in bacterial composition of normal breast and NAT, which should prompt caution in the interpretation of the prior reports of the “normal” breast microbiome. Although the link with BC risk factors emerging from this investigation is unclear, the study reports that factors such as parity, breastfeeding, BC predisposition, age, and racial origin can affect the microbial composition of the breast and thus the gene expression and biology of the tissue. A thorough understanding of the breast microbiome and the impact of host genetics, lifestyle, and socioeconomic factors is critical for identifying both the beneficial and harmful microbiota that may affect BC development and its potential role in prevention and treatment.

## Supplementary Information

**Additional file 1.** Tables including sample annotations and raw data.

**Additional file 2.** Figures showing supplementary data.

## Data Availability

Microbiome data are available in the National Center for Biotechnology Information (NCBI)-Sequence Read Archive (SRA) under the accession number PRJNA867176. Transcriptome data are available in Gene Expression Omnibus (GEO) with accession number GSE205725.
