# Supplementary material for: Exploring breast tissue microbial composition and the association with breast cancer risk factors
Source: Breast Cancer Res. 2023 Jul 10;25:82. doi: 10.1186/s13058-023-01677-6 (PMC10332106; doi:10.1186/s13058-023-01677-6)
Supplement: Supplementary file 2 — Additional file 2. Figures showing supplementary data. [file 13058_2023_1677_MOESM2_ESM.pdf]

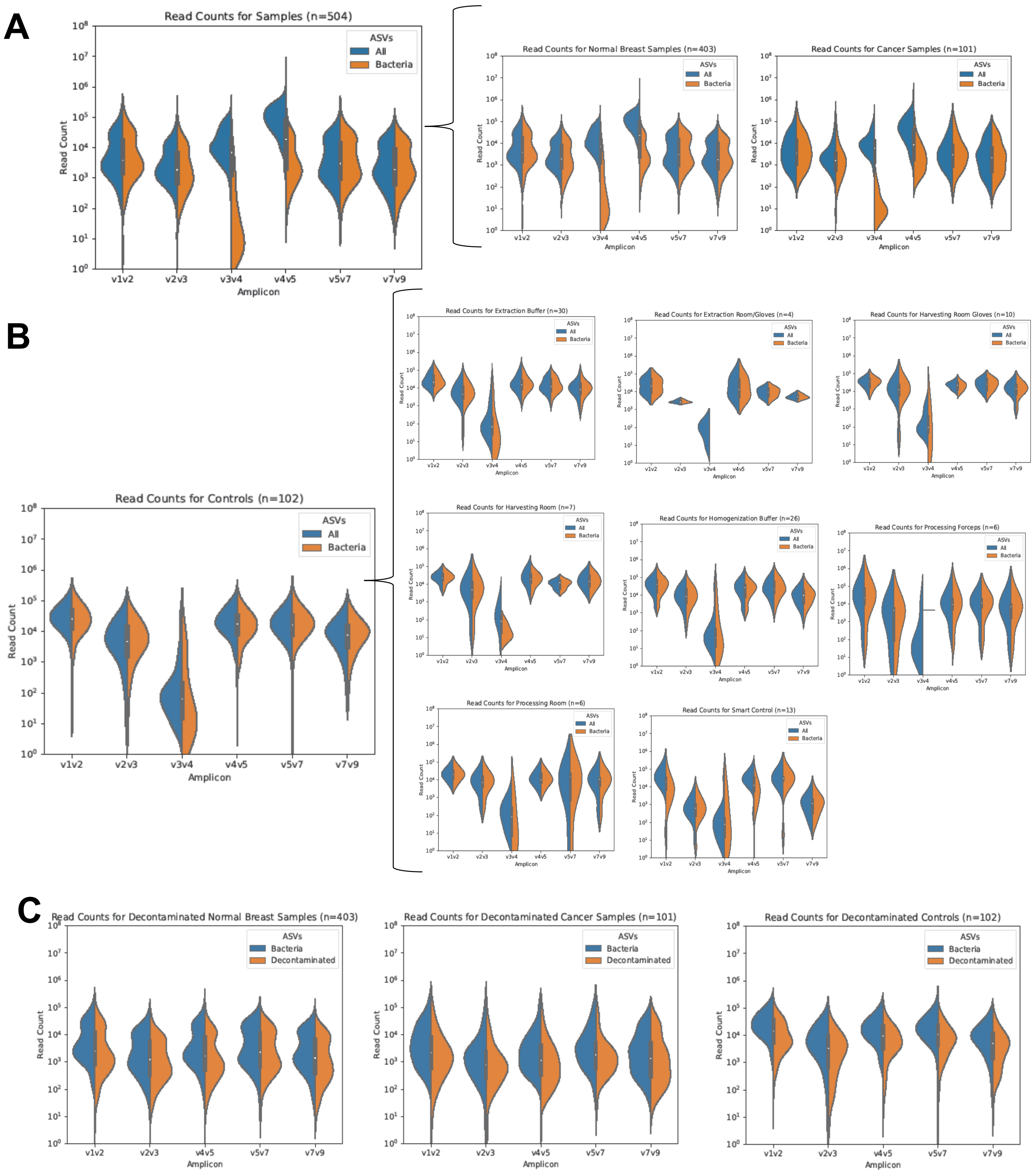

Supplementary Figure S1: Amplicon sequence variance (ASV) abundance in both breast tissue and negative control samples. Violin plots show the distribution of either all (human + bacteria ASV, right blue half of the plot) or bacteria-only (orange left half of the plot) amplicon read count generated via 16s rRNA sequencing of both breast tissue samples (**A**) and several negative controls (**B**) used in this study. **C**) ASV level in samples from healthy (left panel) and tumor bearing women (center panel) and negative controls used in the study (right panel) prior to and after the second step of decontamination via Decon.

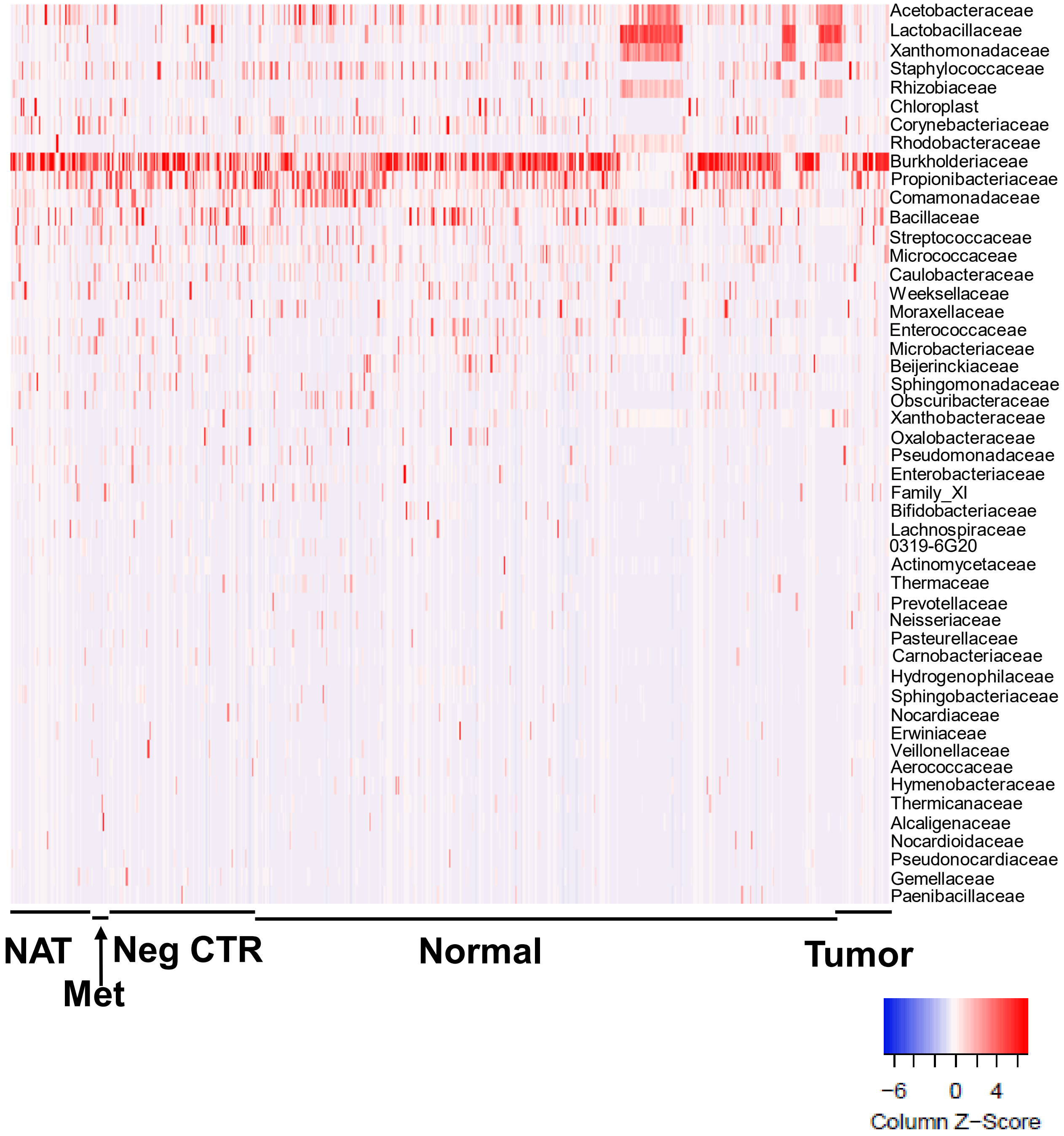

Supplementary Figure S2: Heat Map of the bacterial abundance in breast tissues. Fifty most abundant bacterial families detected in the healthy breast (Normal), normal adjacent to tumor (NAT), Tumor, breast cancer metastasis (Met), and negative control (Neg CTR) samples.

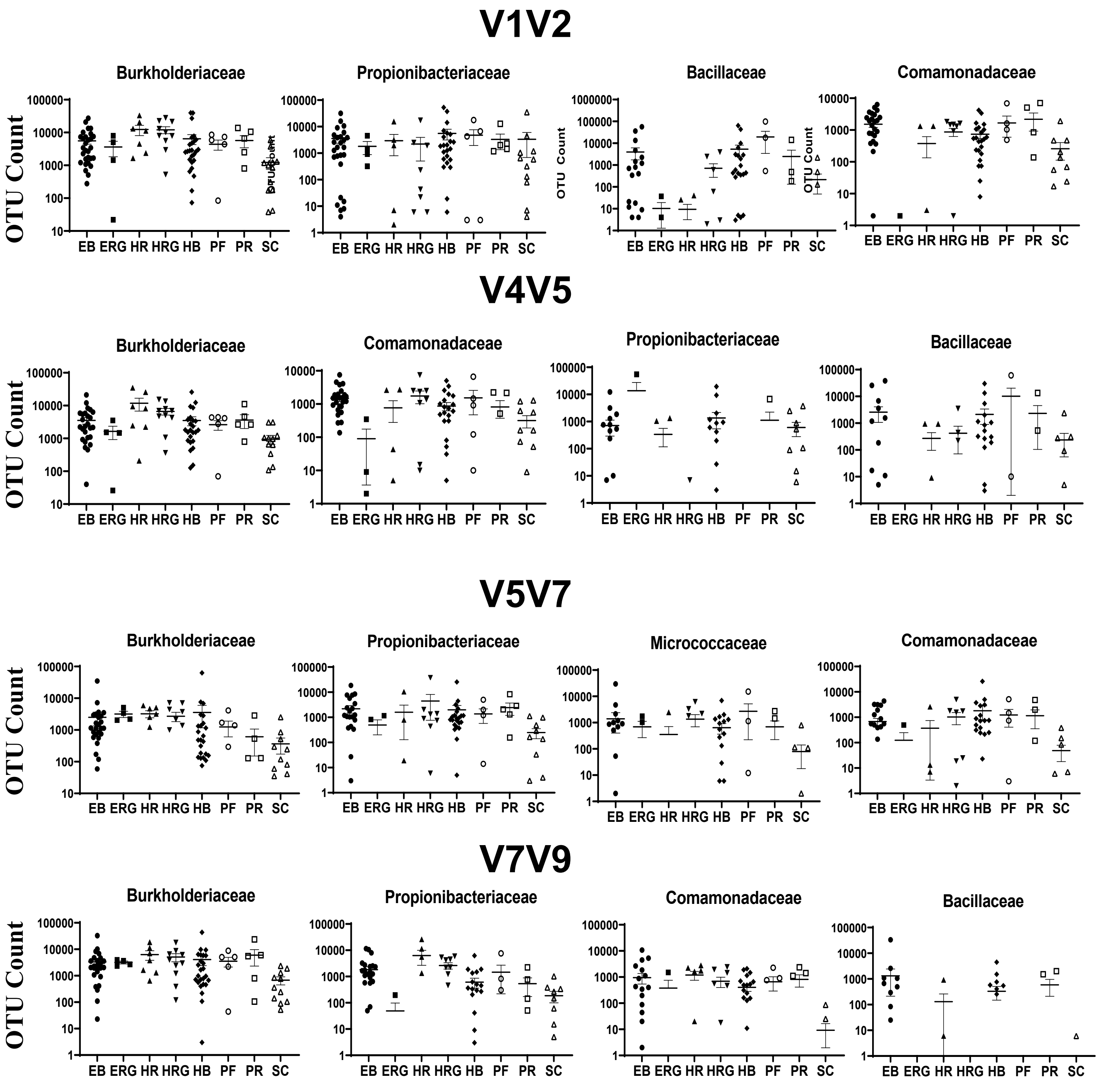

Supplementary Figure S3: Bacterial detection at the family level in negative controls samples. Most abundant bacteria detected via either V1V2, V4V5, V5V7, or V7V9 amplicon analysis. EB: extraction buffer, ERG: extraction room and gloves, HR: harvesting room, HRG: harvesting room gloves, HB: homogenization buffer, PF: processing forceps, PR: processing room, SC: library internal control or smartcontrol.

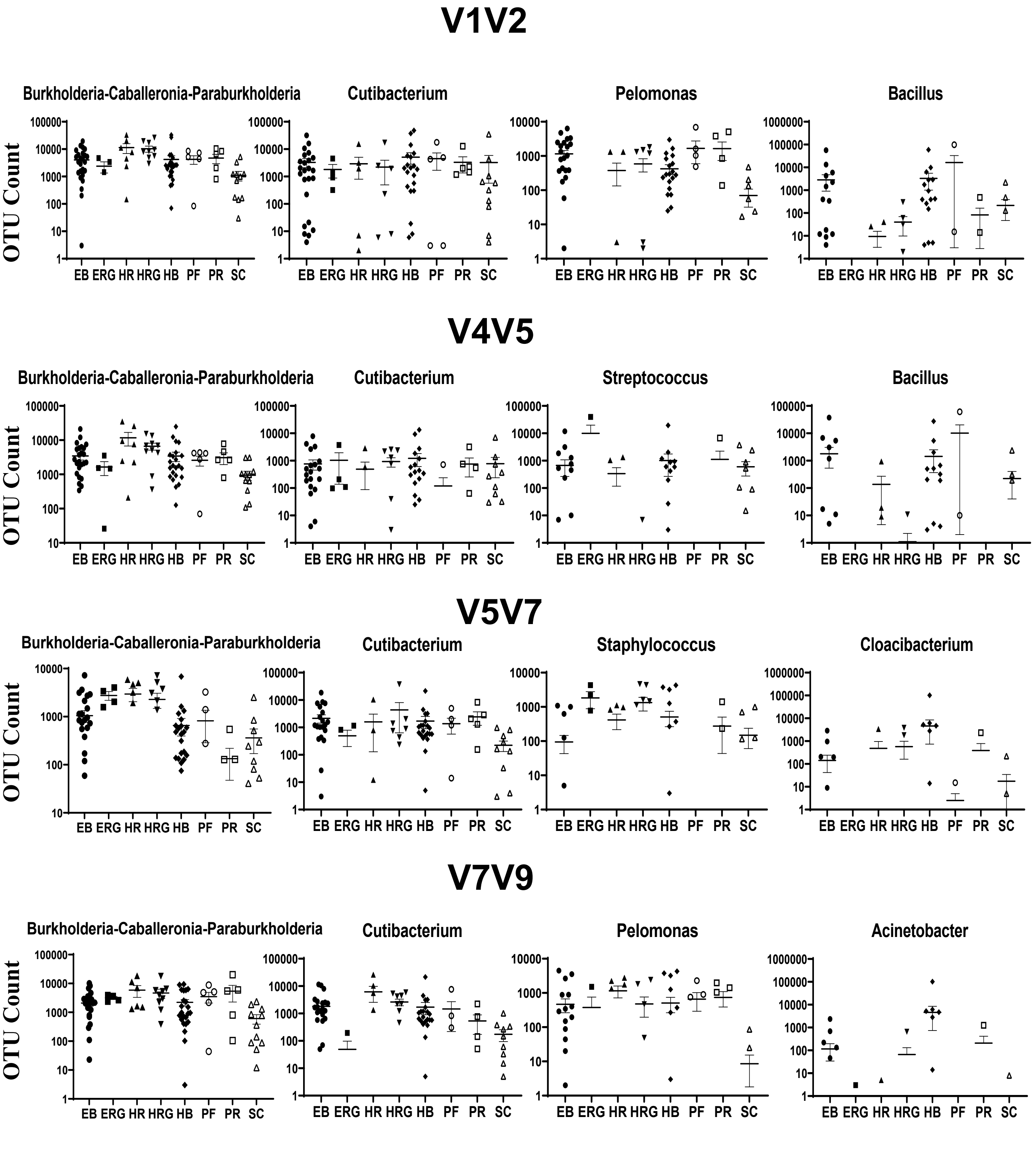

Supplementary Figure S4: Bacterial detection at the genus level in negative controls samples. Most abundant bacteria detected via either V1V2, V4V5, V5V7, or V7V9 amplicon analysis. EB: extraction buffer, ERG: extraction room and gloves, HR: harvesting room, HRG: harvesting room gloves, HB: homogenization buffer, PF: processing forceps, PR: processing room, SC: library internal control or smartcontrol.

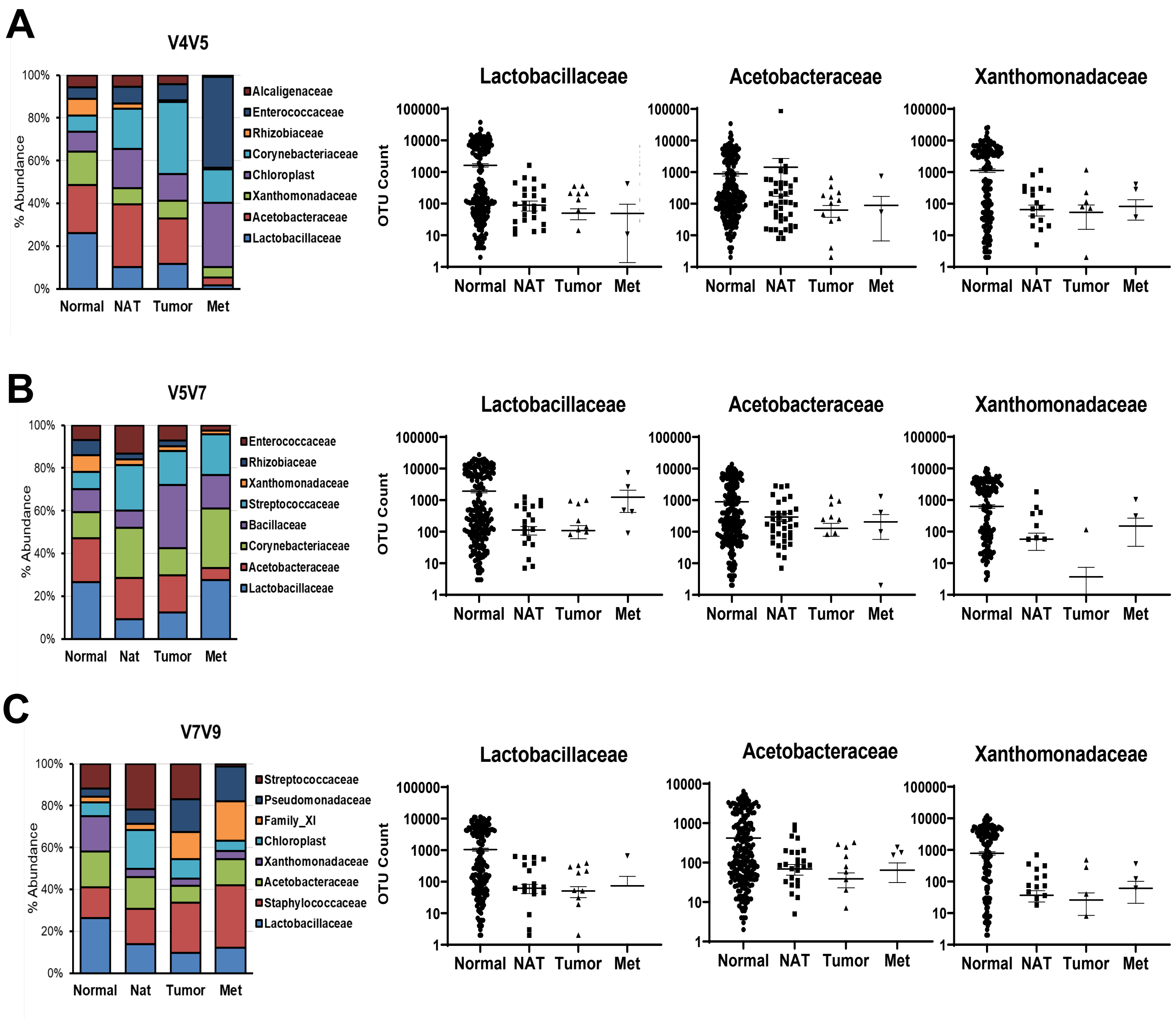

Supplementary Figure S5: Bacterial detection at the family level in breast tissue samples. Most abundant bacteria (>2% abundance) detected via either V4V5, V5V7, or V7V9 amplicon analysis in the healthy breast (Normal), normal adjacent to tumor (NAT), Tumor, breast cancer metastasis (Met), and negative control (Neg CTR) samples. Scatter plots on the right shows the operational taxonomic unit (OTU) count of the three bacterial families most abundant in the normal breast.

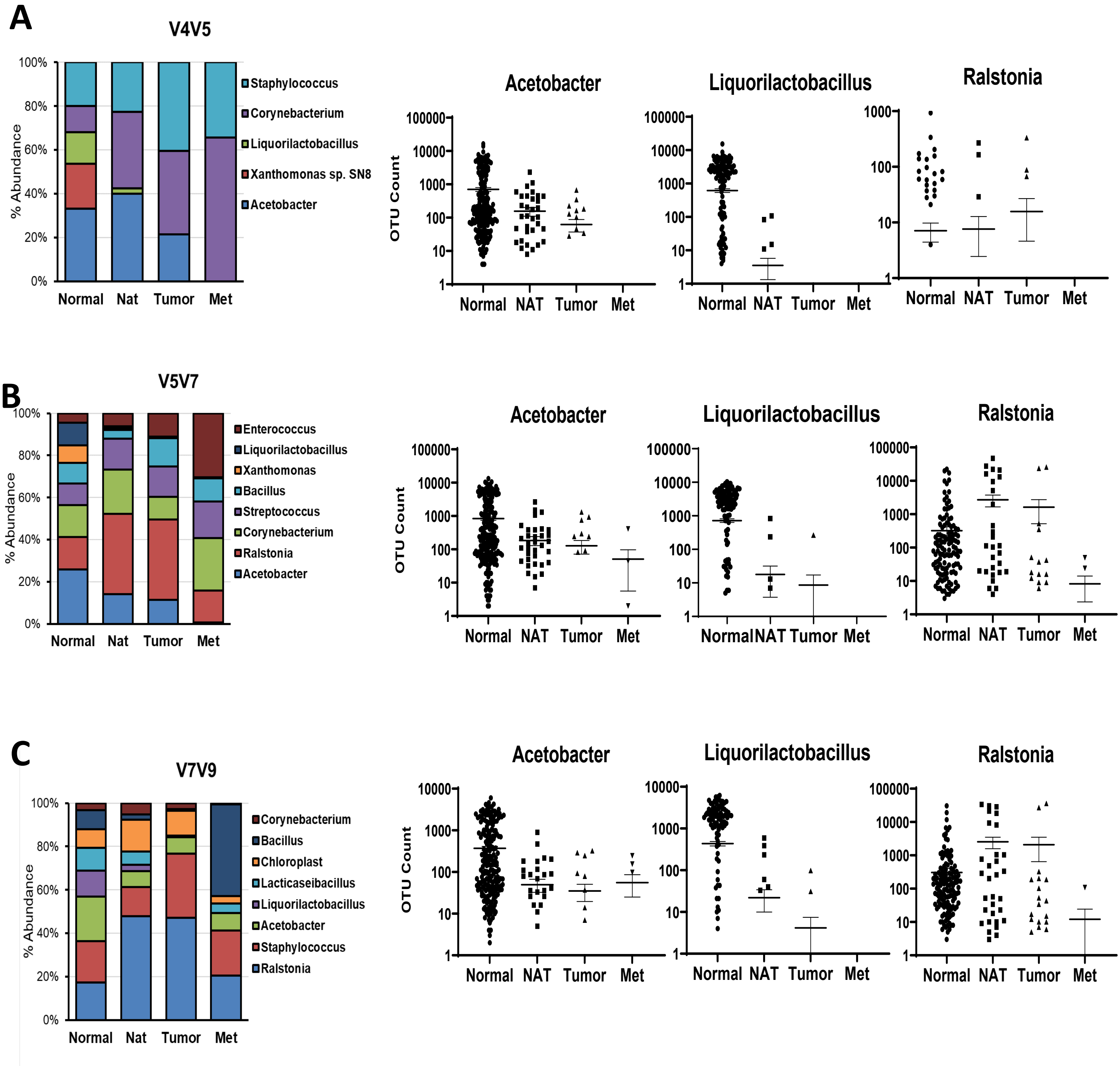

Supplementary Figure S6: Bacterial detection at the genus level in breast tissue samples. Most abundant bacteria (>2% abundance) detected via either V4V5, V5V7, or V7V9 amplicon analysis in the healthy breast (Normal), normal adjacent to tumor (NAT), Tumor, breast cancer metastasis (Met), and negative control (Neg CTR) samples. Scatter plots on the right shows the operational taxonomic unit (OTU) count of the three bacterial genera most abundant in the normal breast.

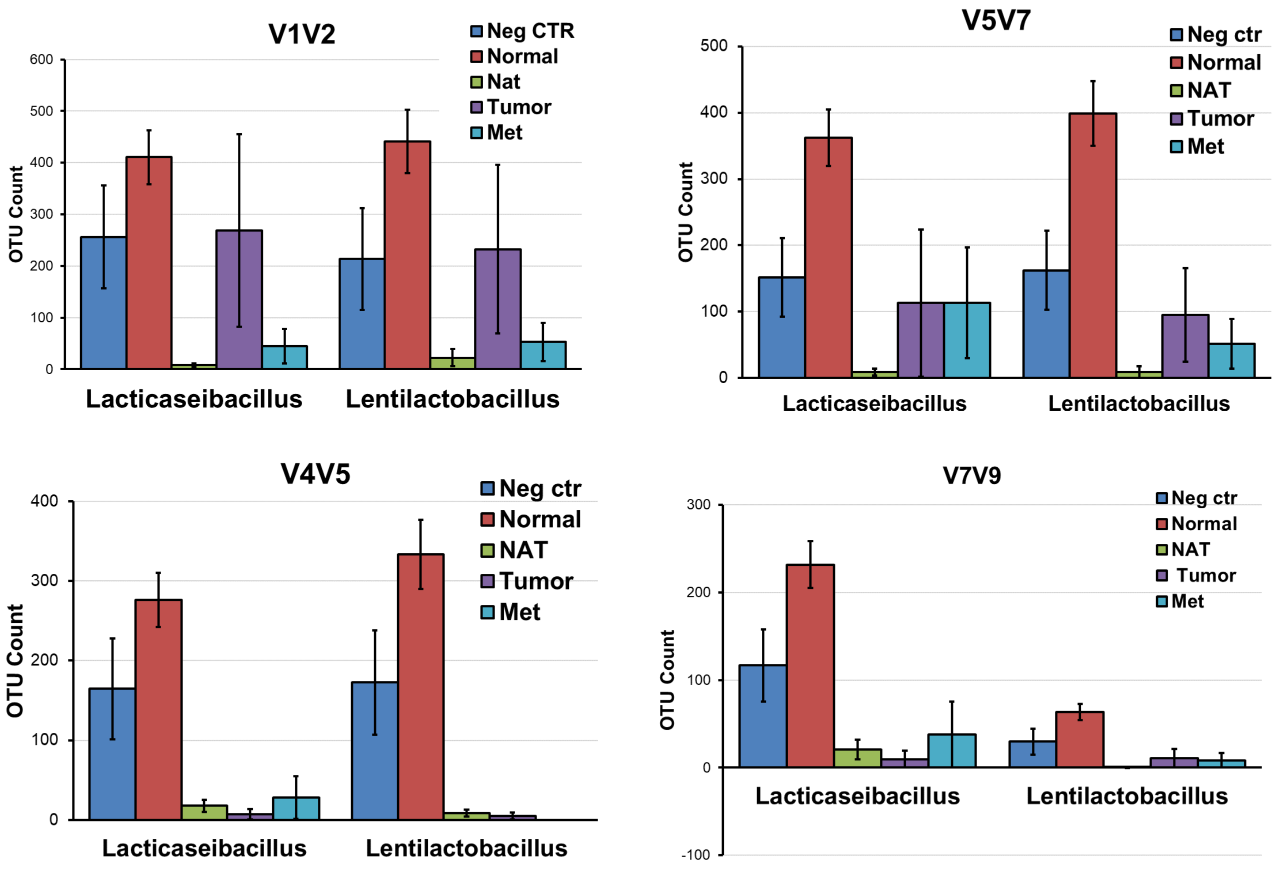

Supplementary Figure S7: Lacticaseibacillus and Lentilactobacillus abundance in breast tissues. Operational taxonomic unit (OTU) count for the two bacterial genera in the healthy breast (Normal), normal adjacent to tumor (NAT), Tumor, breast cancer metastasis (Met), and negative control (Neg CTR) samples. Four different amplicons for 16s-rRNA hypervariable regions were examined.

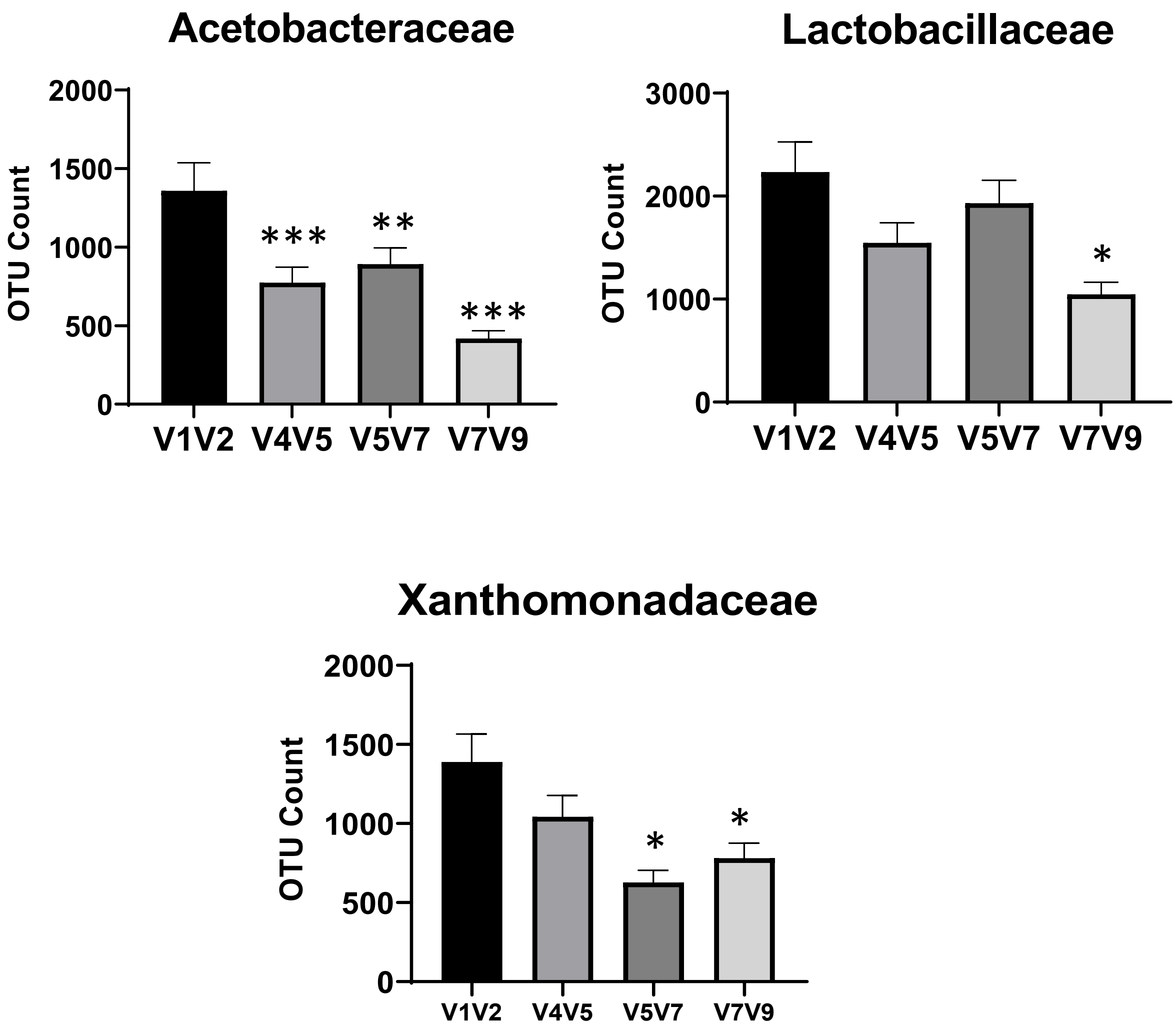

Supplementary Figure S8: Amplicon efficiency in microbial detection in normal breast tissues. Operational taxonomic unit (OTU) count for the three most abundant bacterial families generated by four different 16s-rRNAvamplicons in the Normal samples were examined. *:p<0.05; **:p<0.001; ***:p<0.0001.
